# Baseline Liver Metabolic Health in Newly Diagnosed People Living with HIV Compared with HIV-Negative Controls: Cross-Sectional Findings from the Prospective MASHIV-TR Cohort

**DOI:** 10.3390/ijms27198672

**Published:** 2026-09-28

**Authors:** Çiğdem Mermutluoğlu, Yeşim Taşova, Ayşe Özlem Mete, İlkay Karaoğlan, Hüsnü Pullukçu, Meliha Çağla Sönmezer, Dilara İnan, Funda Şimşek, Sıla Akhan, Tuba Damar Çakırca, Nursel Çalık Başaran, Aslıhan Candevir, Yeliz Çiçek, Damla Ertürk, Ahmet Çağkan İnkaya, İsmail Yıldız, Behice Kurtaran, Müge Toygar Deniz, Yaşar Bayındır, Mustafa Kemal Çelen

**Affiliations:** 1 Department of Infectious Diseases and Clinical Microbiology, Faculty of Medicine, Dicle University, Diyarbakır 21280, Türkiye; cigdem.mermutluoglu@dicle.edu.tr; 2 Department of Infectious Diseases and Clinical Microbiology, Faculty of Medicine, Çukurova University, Adana 01330, Türkiye; ytasova@gmail.com (Y.T.); acandevir@gmail.com (A.C.); damlaerdogan_@hotmail.com (D.E.); 3 Department of Infectious Diseases and Clinical Microbiology, Faculty of Medicine, Gaziantep University, Gaziantep 27310, Türkiye; ayseozlem_ornek@hotmail.com (A.Ö.M.); karaoglan@gantep.edu.tr (İ.K.); 4 Department of Infectious Diseases and Clinical Microbiology, Faculty of Medicine, Ege University, İzmir 35100, Türkiye; husnu.pullukcu@ege.edu.tr; 5 Department of Infectious Diseases and Clinical Microbiology, Faculty of Medicine, Hacettepe University, Ankara 06100, Türkiye; caglasonmezer@hacettepe.edu.tr (M.Ç.S.); inkaya@hacettepe.edu.tr (A.Ç.İ.); 6 Department of Infectious Diseases and Clinical Microbiology, Faculty of Medicine, Akdeniz University, Antalya 07070, Türkiye; inan@akdeniz.edu.tr; 7 Department of Infectious Diseases and Clinical Microbiology, Prof. Dr. Cemil Taşçıoğlu City Hospital, University of Health Sciences Türkiye, İstanbul 34384, Türkiye; fundasimsek67@gmail.com; 8 Department of Infectious Diseases and Clinical Microbiology, Faculty of Medicine, Kocaeli University, Kocaeli 41380, Türkiye; sila.akhan@kocaeli.edu.tr (S.A.); mugedeniz90@gmail.com (M.T.D.); 9 Department of Infectious Diseases and Clinical Microbiology, Faculty of Medicine, Harran University, Şanlıurfa 63300, Türkiye; dr.tubadamar@gmail.com; 10 Department of Internal Medicine, Division of General Internal Medicine, Faculty of Medicine, Hacettepe University, Ankara 06100, Türkiye; nurselc@hacettepe.edu.tr; 11 Epidemiology Doctorate Program, Graduate School of Health Sciences, Istanbul Medipol University, İstanbul 34815, Türkiye; yeliz.cicek@std.medipol.edu.tr; 12 Department of Biostatistics and Medical Informatics, Faculty of Medicine, Dicle University, Diyarbakır 21280, Türkiye; iyildiz@dicle.edu.tr; 13 Department of Infectious Diseases and Clinical Microbiology, Acıbadem Adana Hospital, Adana 01130, Türkiye; behicekurtaran@gmail.com; 14 Department of Infectious Diseases and Clinical Microbiology, Güven Hospital, Ankara 06540, Türkiye; yasarb44@hotmail.com

**Keywords:** HIV, MASLD, vibration-controlled transient elastography, controlled attenuation parameter, liver stiffness measurement, FAST score

## Abstract

Metabolic dysfunction-associated steatotic liver disease (MASLD) is an important comorbidity in people living with HIV (PLWH), yet most data derive from individuals receiving antiretroviral therapy (ART). In the baseline cross-sectional analysis of the prospective multicentre MASHIV-TR cohort (nine centres, Türkiye), we compared 250 newly diagnosed, ART-naïve PLWH and 161 HIV-negative controls. Hepatic steatosis and liver stiffness were assessed by vibration-controlled transient elastography; MASLD was defined as hepatic steatosis, indicated by CAP ≥ 248 dB/m together with at least one cardiometabolic risk factor. MASLD prevalence was similar in the two groups (25.2% vs. 30.4%; *p* = 0.245), and HIV status was not independently associated with MASLD. Triglyceride level was independently associated with CAP in PLWH (+10.69 dB/m per 50 mg/dL) and in controls (+9.26 dB/m), whereas CD4 and CD8 counts, the CD4/CD8 ratio and HIV-RNA were not associated with the liver outcomes. Before ART, hepatic steatosis tracked metabolic rather than HIV-specific markers.

## 1. Introduction

Metabolic dysfunction-associated steatotic liver disease (MASLD; formerly termed nonalcoholic fatty liver disease) is a growing global public health concern, affecting approximately 38% of adults worldwide and increasing in parallel with the rising prevalence of obesity and type 2 diabetes. The disease spans a broad clinical spectrum, from simple steatosis to metabolic dysfunction-associated steatohepatitis (MASH; formerly termed nonalcoholic steatohepatitis), which is characterised by inflammation and hepatocellular injury, and further to progressive fibrosis, cirrhosis, and hepatocellular carcinoma. Although MASLD does not progress to advanced liver disease in every patient, cardiovascular disease remains the leading cause of death in affected individuals; the condition is also associated with an increased risk of incident type 2 diabetes, chronic kidney disease, sarcopenia, and extrahepatic malignancies. Projections that the prevalence of MASLD among adults will exceed 55% by 2040 suggest that its clinical and economic burden may increase further [1,2].

Although hepatic involvement in people living with HIV (PLWH) was historically attributed primarily to hepatitis B virus and hepatitis C virus coinfections, MASLD has emerged as an increasingly recognised and important chronic liver disease in this population. Through chronic immune activation, microbial translocation, and persistent inflammation, HIV infection may exert systemic effects that promote oxidative stress, hepatic inflammation, and fibrogenesis [3,4]. Early initiation of contemporary, highly effective antiretroviral therapy (ART) enables rapid virological suppression and immune restoration, thereby substantially reducing HIV-related morbidity and mortality [5,6]. Nevertheless, some ART regimens are associated with metabolic changes such as weight gain, insulin resistance, and dyslipidaemia, which may contribute to the development of MASLD [7,8,9,10,11,12,13]. In recent studies, the reported prevalence of MASLD among PLWH has varied according to the diagnostic method used and the characteristics of the study population. A systematic review and meta-analysis reported an NAFLD prevalence of 33.9% among PLWH in imaging-based studies [14]. Studies using vibration-controlled transient elastography (VCTE) have reported a MASLD prevalence of approximately 26.6% among PLWH, whereas a large cohort study in the United States reported a prevalence of 39% [15,16]. Differences across studies may be related not only to the controlled attenuation parameter (CAP) thresholds used but also to the demographic and metabolic characteristics of the participants. A recent scoping review of 66 studies reported hepatic steatosis in approximately 49% of PLWH by ultrasonography and 51% by CAP [17].

The clinical impact of MASLD is also reflected in its physical and psychosocial consequences. Individuals with MASLD have been reported to experience reduced health-related quality of life, with fatigue, depressive symptoms, physical limitations, and impaired daily functioning occurring more frequently than in individuals without MASLD [18,19]. Therefore, the evaluation of MASLD in PLWH should consider not only biochemical and liver-related indicators but also the potential effects of the disease on quality of life and functional status.

MASLD is often asymptomatic and may be overlooked during routine clinical evaluation. Normal alanine aminotransferase (ALT) and aspartate aminotransferase (AST) levels do not exclude the disease or advanced fibrosis. Current guidelines therefore recommend a stepwise approach that incorporates the metabolic risk profile and applies blood-based noninvasive fibrosis scores followed by elastography, rather than relying solely on serum transaminase levels [20,21]. Consistent with this, Michel et al. reported that ALT and AST levels were within the normal range in a substantial proportion of individuals diagnosed with MASLD [15]. This finding underscores the complementary role of noninvasive methods alongside biochemical tests in assessing liver health among PLWH.

Because liver biopsy has limited use in routine clinical practice, noninvasive tests have become central to the assessment of hepatic steatosis and fibrosis [22]. VCTE is a reliable and reproducible method that is widely used because it noninvasively assesses hepatic steatosis using CAP and liver fibrosis using liver stiffness measurement (LSM) [23]. The FibroScan-AST (FAST) score, which combines VCTE parameters with serum AST levels, is a validated algorithm that estimates the likelihood of active fibrotic steatohepatitis without biopsy [24]. Although ultrasonography- and magnetic resonance-based imaging modalities can also assess steatosis, they have method-specific limitations in accessibility and cost [20,21,25,26].

Despite the rapidly expanding global literature on MASLD among PLWH, multicentre studies prospectively evaluating liver health in Türkiye, particularly among newly diagnosed individuals who have not yet initiated ART, remain limited. Consequently, data on the metabolic profile, the burden of hepatic steatosis, and associated risk factors at the time of HIV diagnosis are insufficient. This study aimed to determine the prevalence of MASLD among individuals recently diagnosed with HIV and not yet receiving ART in Türkiye. It also aimed to identify the clinical and metabolic factors associated with MASLD at diagnosis. Two features of the Turkish setting frame this question. First, the national burden of MASLD is high. A recent multicentre study using VCTE with the steatosis threshold applied here (CAP ≥ 248 dB/m) reported a prevalence of 57.5% among adults with at least one cardiometabolic risk factor [27]. Ultrasonography-based and histological series have reported prevalences of 45.5% and 43.3%, respectively [28,29]. Second, the Turkish HIV epidemic is strongly male-predominant. National surveillance data indicate that about 81% of all notified HIV cases, and 85% of those notified in the most recent reporting year, were male. The male-to-female ratio is 5.39, compared with 1.8 for the WHO European Region [30]. Any comparison of newly diagnosed PLWH with HIV-negative individuals in this setting must therefore address a pronounced sex imbalance; we addressed this analytically rather than by matching.

## 2. Results

The participant flow and the analysed sample for each outcome are shown in Figure 1. The baseline analyses included 411 participants: 250 newly diagnosed, ART-naïve PLWH and 161 HIV-negative controls. The median age was 34.0 (28.0–44.0) years in the PLWH group and 30.0 (27.0–43.0) years in the control group (*p* = 0.023; SMD = 0.22). The proportion of men was higher in the PLWH group (90.8% vs. 57.1%; *p* < 0.001; SMD = 0.83). Educational attainment also differed between the groups (*p* < 0.001; SMD = 0.39); the proportion of university graduates was higher among controls, whereas primary- and middle-school graduates were more frequent among PLWH.

BMI was lower in the PLWH group than in the control group (24.24 [22.21–27.64] vs. 26.23 [23.66–28.09] kg/m^2^; *p* < 0.001; SMD = 0.43). Body weight, waist circumference, and waist-to-hip ratio did not differ significantly between the groups. Hip circumference was lower among PLWH, although the absolute difference was small (98.0 [93.0–104.0] vs. 99.0 [95.0–106.0] cm; *p* = 0.042; SMD = 0.20).

At least one comorbidity was more common in the PLWH group (31.6% vs. 15.5%; *p* < 0.001; SMD = 0.39). Coronary artery disease was observed only among PLWH (*p* = 0.001; SMD = 0.37), whereas diabetes mellitus and hypertension were similarly prevalent in the two groups. Current smoking was more frequent among PLWH (55.2% vs. 40.4%; *p* = 0.003; SMD = 0.30). Alcohol use and physical activity did not differ between the groups. In contrast, the distribution of lifestyle patterns differed (*p* < 0.001): a regular lifestyle was more frequent among PLWH (47.6% vs. 27.3%), whereas an intensive work schedule was less common among PLWH (13.2% vs. 42.2%). A sedentary lifestyle was similarly prevalent in the two groups (34.8% vs. 30.4%).

With respect to glucose metabolism, fasting plasma glucose, fasting insulin, and HOMA-IR were higher in the PLWH group (*p* < 0.001, *p* = 0.038, and *p* = 0.002, respectively), whereas HbA1c levels were similar between the groups (*p* = 0.272). Triglyceride levels were lower among PLWH (140.0 [108.0–177.0] vs. 177.0 [121.0–211.0] mg/dL; *p* < 0.001; SMD = 0.31), whereas total cholesterol and non-HDL cholesterol levels were higher (*p* = 0.018 and *p* = 0.012, respectively). HDL- and LDL-cholesterol levels did not differ significantly between the groups.

Platelet and leukocyte counts were higher in the PLWH group (*p* ≤ 0.025 for both). ALT levels were lower among PLWH, although the absolute difference was small (23.0 [16.0–31.0] vs. 26.0 [19.75–46.0] U/L; *p* = 0.018; SMD = 0.20). No significant differences were observed in the other haematological or biochemical parameters.

Among PLWH, the median CD4+ T-cell count was 371.5 (240.0–574.0) cells/µL, the median CD8+ T-cell count was 962.0 (709.3–1400.0) cells/µL, and the median CD4/CD8 ratio was 0.39 (0.22–0.60). The median HIV RNA level was 93,700 (32,500–326,900) copies/mL, and the median log10 HIV RNA level was 4.97 (4.51–5.51) log10 copies/mL. The demographic, clinical, and laboratory characteristics of the participants are presented in Table 1.

Among the noninvasive measures of liver health, only CAP differed between the groups. Median CAP was lower in the PLWH group than in the control group (217.0 [199.0–248.3] vs. 228.0 [201.0–256.0] dB/m; *p* = 0.030). The groups did not differ significantly in LSM (5.30 [4.40–6.70] vs. 5.30 [4.40–6.60] kPa; *p* = 0.624), FIB-4 (0.78 [0.54–1.19] vs. 0.82 [0.59–1.17]; *p* = 0.488), or FAST score (0.10 [0.06–0.19] vs. 0.13 [0.07–0.22]; *p* = 0.065; Table 2 and Supplementary Table S1). FAST-based analyses included 250 PLWH and 158 controls because serum AST was unavailable in three controls. The distributions of CAP and LSM in the two groups are shown in Figure 2.

The prevalence of MASLD was similar in the PLWH and HIV-negative control groups (25.2% vs. 30.4%; *p* = 0.245; SMD = 0.12). The proportions of participants with FAST ≥ 0.35 (8.0% vs. 8.9%; *p* = 0.759) and LSM ≥ 8 kPa (12.4% vs. 19.3%; *p* = 0.058) also did not differ between the groups (Table 2; Supplementary Table S1). Unadjusted logistic regression analyses yielded similar findings for MASLD, FAST ≥ 0.35, and LSM ≥ 8 kPa (Table 3; Supplementary Table S1). The prevalences of the binary liver outcomes in both groups are shown in Figure 3.

In Model 1 (adjusted for age and sex) and Model 2 (adjusted for age, sex and body mass index), HIV status was not independently associated with MASLD, FAST ≥ 0.35, or LSM ≥ 8 kPa. Analyses of LSM as both a continuous measure and a binary outcome confirmed this finding. In contrast, the nominal differences observed for CAP, FAST, and FIB-4 were not consistently confirmed in the sensitivity analyses. The propensity-score model additionally included educational attainment, comorbidity, smoking, physical activity, sleep duration, lifestyle and dietary pattern, alcohol use, and triglyceride level. Details of the adjusted and sensitivity analyses are presented in Table 3 and Supplementary Table S1.

Propensity-score overlap weighting reduced the absolute standardised mean difference for every covariate in the model to <0.001, including sex (0.337 before weighting), reflecting the exact mean-balance property of overlap weights [31]. The effective sample size was 95.5 for PLWH and 76.6 for controls (Supplementary Tables S2 and S3). In this exactly balanced cohort, HIV status remained unassociated with MASLD (OR 0.75; 95% CI, 0.39–1.44), CAP (−8.41 dB/m; 95% CI, −21.08 to 4.27), LSM (0.33 kPa; 95% CI, −0.49 to 1.15), LSM ≥ 8 kPa (OR 0.65; 95% CI, 0.29–1.46) or FAST ≥ 0.35 (OR 0.84; 95% CI, 0.33–2.14). Sex, however, modified the association between HIV status and MASLD (interaction OR 0.12; 95% CI, 0.04–0.37; *p* < 0.001). MASLD was present in 52 of 227 men (22.9%) and 11 of 23 women (47.8%) in the PLWH group, compared with 36 of 92 men (39.1%) and 13 of 69 women (18.8%) among controls. In the male-only subgroup, MASLD was significantly less frequent among PLWH than among controls (22.9% vs. 39.1%; OR 0.46; *p* = 0.005), whereas LSM ≥ 8 kPa (12.8% vs. 17.4%; OR 0.70; *p* = 0.291) and FAST ≥ 0.35 (8.4% vs. 8.8%; OR 0.95; *p* = 1.000) did not differ. After direct standardisation of the control group to the sex distribution of the PLWH group, the standardised MASLD prevalence among controls rose from 30.4% to 37.3%. The standardised prevalences of LSM ≥ 8 kPa (17.8%) and FAST ≥ 0.35 (8.8%) were essentially unchanged. Excluding the five control examinations with an IQR-to-median ratio above 0.30 did not alter the primary comparison (MASLD 25.2% vs. 31.4%; OR 0.74; *p* = 0.209). Estimates for HIV status across all adjustment strategies are shown in Figure 4, and the sex-specific prevalences in Figure 5.

Among PLWH, CAP correlated positively with triglyceride levels (ρ = 0.40; FDR-adjusted *p* < 0.001), LDL cholesterol (ρ = 0.28; FDR-adjusted *p* < 0.001), fasting plasma glucose (ρ = 0.24; FDR-adjusted *p* = 0.001), and BMI (ρ = 0.19; FDR-adjusted *p* = 0.017), and inversely with HDL cholesterol (ρ = −0.28; FDR-adjusted *p* < 0.001) and the HDL/LDL-cholesterol ratio (ρ = −0.41; FDR-adjusted *p* < 0.001). A weak positive correlation was also observed with GGT (ρ = 0.17; FDR-adjusted *p* = 0.039). CAP was not significantly correlated with CD4 cell count, CD8 cell count, CD4/CD8 ratio, or log10 HIV RNA level (Table 4).

In univariable analyses, triglyceride, LDL-cholesterol, and ALT levels were positively associated with MASLD, whereas HDL-cholesterol levels were inversely associated. Male sex was inversely associated with MASLD (OR 0.33; 95% CI, 0.14–0.78; *p* = 0.012) and BMI was positively associated with MASLD, but neither association remained significant after FDR correction. Similarly, alcohol use and HIV-specific variables (CD4 cell count, CD8 cell count, CD4/CD8 ratio, and log10 HIV RNA level) were not significantly associated with MASLD (Table 4).

The FAST score correlated positively with ALT (ρ = 0.33; FDR-adjusted *p* < 0.001) and triglyceride levels (ρ = 0.21; FDR-adjusted *p* = 0.021). In univariable analysis, only ALT was significantly associated with FAST ≥0.35; the nominal associations observed for triglycerides, CD4 cell count, and HIV RNA did not remain significant after FDR correction. Among PLWH, FAST scores were higher in participants with MASLD than in those without (*p* = 0.003), whereas in the control group they did not differ according to MASLD status (*p* = 0.269). Detailed results are presented in Supplementary Table S1.

In multivariable analyses, only triglyceride levels were independently associated with both CAP and MASLD. Each 50 mg/dL increase in triglyceride levels was associated with a 10.69 dB/m increase in median CAP (95% CI, 5.51–15.88; FDR-adjusted *p* < 0.001). Similarly, each 50 mg/dL increase in triglyceride levels was associated with higher odds of MASLD (adjusted OR, 1.70; 95% CI, 1.32–2.18; FDR-adjusted *p* < 0.001). Although BMI, male sex, and alcohol use showed nominal associations, these did not remain significant after FDR correction. The nominal association between HIV RNA level and continuous FAST score also did not remain significant after FDR correction. Overall, no HIV-specific variable was independently associated with CAP, LSM, MASLD, or FAST. Details of the multivariable analyses are presented in Table 4 and Supplementary Table S1.

In the HIV-negative control group, univariable mean bias-reduced logistic regression analyses showed that male sex, HOMA-IR, triglyceride levels, and alcohol use were positively associated with MASLD, whereas HDL-cholesterol levels were inversely associated with MASLD. These findings remained significant after FDR correction. In contrast, age, BMI, waist circumference, hip circumference, waist-to-hip ratio, fasting plasma glucose, fasting insulin, HbA1c, total cholesterol, LDL cholesterol, non-HDL cholesterol, ALT, AST, GGT, current smoking, and the presence of comorbidity were not significantly associated with MASLD (Table 5).

In the multivariable model that jointly evaluated metabolic variables, only triglyceride levels remained independently associated with MASLD (adjusted OR per 50 mg/dL increase, 1.66; 95% CI, 1.13–2.44; FDR-adjusted *p* = 0.041). HDL-cholesterol levels showed a nominal inverse association, but it did not remain significant after within-model FDR correction. BMI and HOMA-IR were not independently associated with MASLD (Table 5).

In the sensitivity analysis that jointly included age, sex, triglycerides, and HDL cholesterol, male sex, triglyceride levels, and HDL-cholesterol levels retained their independent associations with MASLD, whereas age showed no independent association. The same pattern was observed in the sensitivity analysis using the recorded MASLD variable (Table 5).

Analyses addressing the definitional overlap between the exposure and the outcome are summarised below. Every participant with hepatic steatosis met at least one cardiometabolic criterion (63 of 63 PLWH and 49 of 49 controls). The cardiometabolic requirement was therefore non-binding, and in this cohort MASLD status was determined by CAP alone. Among steatotic participants, 7 of 63 PLWH (11.1%) and 4 of 49 controls (8.2%) met the cardiometabolic requirement exclusively through the triglyceride and/or HDL-cholesterol criterion, whereas the remainder also satisfied a non-lipid criterion. When MASLD was redefined using only the non-lipid criteria, triglyceride level remained independently associated with MASLD in PLWH (adjusted OR per 50 mg/dL, 1.61; 95% CI, 1.23–2.10; *p* < 0.001) and in controls (adjusted OR, 1.50; 95% CI, 1.04–2.16; *p* = 0.031), closely matching the estimates obtained with the standard definition. Triglyceride level was also independently associated with continuous CAP among controls (adjusted median difference 9.26 dB/m per 50 mg/dL; 95% CI, 2.48–16.62; ρ = 0.26; *p* < 0.001), mirroring the association observed among PLWH. The recalculated MASLD classification agreed with the classification originally recorded at the participating centres in 87.6% of the 411 participants (51 discordant cases). Repeating the primary models with the originally recorded classification did not alter the direction or significance of the triglyceride association. The relationship between triglyceride level and CAP in both groups is shown in Figure 6.

## 3. Discussion

Using baseline data from the MASHIV-TR study, we compared the prevalence of MASLD between newly diagnosed, ART-naïve PLWH and HIV-negative controls and evaluated the metabolic and HIV-specific determinants associated with MASLD. The prevalence of MASLD was similar in the two groups. Metabolic indicators, however, showed stronger associations with hepatic steatosis than HIV-specific immunological and virological markers did. In multivariable analyses, triglyceride levels remained independently associated with both CAP values and MASLD among PLWH and were also independently associated with MASLD among HIV-negative controls. By contrast, CD4 cell count, the CD4/CD8 ratio, and HIV RNA levels showed no independent associations. The independent association of triglyceride levels with both MASLD and CAP values among PLWH, together with the similar association in HIV-negative controls, suggests that hepatic steatosis in newly diagnosed, ART-naïve individuals may be more closely related to metabolic processes than to HIV-specific immunological or virological factors at this early stage. Two qualifications shape the interpretation of these findings. First, the overall absence of a between-group difference in MASLD prevalence concealed opposite sex-specific patterns. Among men, who accounted for 90.8% of the PLWH group, MASLD was significantly less frequent in PLWH than in controls. Among the 23 women in the PLWH group, the direction was reversed. Second, triglyceride level is itself one of the cardiometabolic criteria defining MASLD. In this cohort, however, every participant with hepatic steatosis met the cardiometabolic requirement, so MASLD status was determined by CAP alone, and the association is not an arithmetic consequence of the definition.

Much of the literature on MASLD prevalence among PLWH uses the definition of nonalcoholic fatty liver disease (NAFLD), the term used before the condition was renamed MASLD. A 2025 systematic review and meta-analysis combining these studies reported a steatotic liver disease prevalence of 39% (95% CI, 33–44%) among PLWH, highlighting the substantial burden of metabolic liver disease in this population [32]. Reported estimates of steatosis prevalence among PLWH vary considerably according to the characteristics of the study population, the burden of metabolic risk, exposure to ART, and the method used to assess steatosis. In a European multicentre study by Lemoine et al., magnetic resonance imaging–proton density fat fraction (MRI-PDFF) identified NAFLD in 64% of PLWH at risk of the condition, who had a median age of 55 years, a median BMI of 27.0 kg/m^2^, and at least five years of ART exposure [33]. In the Multicenter AIDS Cohort Study, by contrast, computed tomography-detected hepatic steatosis was less prevalent among PLWH than among HIV-negative controls in a predominantly ART-treated male population with a median age of 53 years (13% vs. 19%; *p* = 0.02) [34]. In that study, the associations of steatosis with visceral adiposity and insulin resistance underscored the importance of metabolic factors irrespective of HIV status. These between-study differences are particularly relevant to Türkiye and the Middle East and North Africa (MENA) region, where the burden of MASLD is increasing rapidly. Rising rates of obesity, type 2 diabetes, and other cardiometabolic risk factors are among the main contributors to this growing regional burden [1]. Nevertheless, data specifically addressing MASLD among PLWH in this region remain limited, particularly from studies comparing PLWH with HIV-negative individuals before ART initiation. The absence of a difference in MASLD prevalence between the groups in our study may be related to the relatively young age of our cohort and the lower BMI in the PLWH group. These characteristics may partly explain why our findings differ from the stronger anthropometric associations reported in previous studies [32,35].

MASLD was accompanied by similar metabolic characteristics in both groups. The parallel patterns observed in biochemical markers of glucose and lipid metabolism suggest that hepatic steatosis in newly diagnosed, ART-naïve PLWH may be more closely associated with conventional cardiometabolic risk factors than with mechanisms specific to HIV infection. These findings are consistent with previous studies showing that MASLD in PLWH is closely linked to metabolic abnormalities [16,35]. Associations with anthropometric measures were more limited. Although BMI was positively associated with both CAP and MASLD in the PLWH group, these associations did not remain significant after FDR correction. BMI was also not independently associated with MASLD in the control group, and no consistent associations were identified for waist circumference, hip circumference, or the waist-to-hip ratio. The strength of the relationship between anthropometric measures and MASLD may vary with the demographic and metabolic characteristics of the study population. A meta-analysis of European studies found that both BMI and waist circumference were significantly associated with NAFLD, whereas a recent multicentre study reported that waist circumference had the highest discriminatory performance for steatosis defined by CAP ≥ 248 dB/m, followed by BMI and hip circumference [36,37]. The weaker associations between anthropometric measures and MASLD in our study may be partly explained by the older age, higher BMI, and greater burden of metabolic comorbidities among participants in those studies. Thus, although the direction of the associations between BMI and both CAP and MASLD in the PLWH group was consistent with previous findings, the independent contribution of BMI remained limited.

In the present study, triglyceride levels were the most consistent metabolic factor associated with liver outcomes in both groups. Among individuals with HIV, elevated triglyceride levels were independently associated with both CAP and MASLD. A comparable independent association with MASLD was observed among HIV-negative controls. By contrast, the associations of HDL cholesterol and LDL cholesterol with MASLD were less consistent and did not persist across all multivariable models. Because elevated triglyceride and low HDL-C levels are among the cardiometabolic criteria used to define MASLD, associations with the binary MASLD outcome should be interpreted with caution. Nevertheless, the independent association between triglyceride levels and continuous CAP values among PLWH suggests that this finding cannot be explained solely by the operational definition of MASLD. Previous studies have also reported an association between elevated triglyceride levels and hepatic steatosis, as assessed by CAP or MRI-PDFF, among PLWH [38]. This finding is further supported by a recent meta-analysis of 17 studies including 3933 PLWH, in which MASLD was associated with a more atherogenic lipid profile, with hypertriglyceridaemia emerging as the most consistent lipid abnormality [39]. Hepatic triglyceride accumulation is linked to increased de novo lipogenesis driven by insulin resistance, increased flux of free fatty acids from adipose tissue to the liver, and the inability of very-low-density lipoprotein (VLDL)-mediated lipid export to accommodate the hepatic lipid burden [40]. Studies in PLWH likewise support a close association of elevated triglyceride levels and other metabolic abnormalities with hepatic steatosis [16,41]. Although the pooled comparisons in the meta-analysis primarily reflect phenotypic differences between individuals with and without MASLD, our multivariable findings extend this evidence by suggesting that triglyceride metabolism may represent a shared metabolic basis of hepatic fat accumulation rather than an HIV-specific process. The metabolic and HIV-related pathways relevant to hepatic steatosis in this cohort, and those that remain to be examined after ART initiation, are summarised in Figure 7.

Insulin resistance is one of the key mechanisms linking hepatic lipid accumulation to systemic metabolic dysfunction in the development of MASLD. In the control group, HOMA-IR was associated with MASLD in univariable analysis (OR 1.37 per unit increase; 95% CI, 1.08–1.75; FDR-adjusted *p* = 0.049), although this association did not persist after adjustment for other metabolic variables (OR 1.26; *p* = 0.082). Among PLWH, HOMA-IR was not associated with CAP (ρ = 0.12; FDR-adjusted *p* = 0.201) or with MASLD (OR 1.04; 95% CI, 0.91–1.19; *p* = 0.547) in any analysis. The association observed in the control group is consistent with previous evidence supporting the role of insulin resistance in the development of hepatic steatosis. Experimental and clinical studies have shown that reduced hepatic and systemic insulin sensitivity is associated with increased de novo lipogenesis and intrahepatic triglyceride accumulation [42]. Similarly, HOMA-IR has been reported to be associated with hepatic steatosis among PLWH [43]. In our data, however, the association between HOMA-IR and MASLD did not remain independent in multivariable analyses. This may indicate that insulin resistance is not an isolated determinant of MASLD but rather part of a complex process that coexists with other metabolic abnormalities.

The two study groups showed different patterns for sex and alcohol exposure. In the PLWH group, male sex was inversely associated with MASLD, although this association did not remain significant after FDR correction. This pattern contrasts with recent meta-analyses identifying male sex as a risk factor for NAFLD/MASLD among PLWH: a meta-analysis of European studies found male sex to be an independent risk factor, and a more recent meta-analysis reported approximately 57% higher odds of MASLD among men [37,39]. However, the predominantly male composition of our PLWH group may have limited our ability to assess sex-related effects reliably. In the control group, male sex was associated with a higher likelihood of MASLD, and this finding persisted in sensitivity analyses. Because the control group was not matched by sex, however, this result may have been influenced by differences in group composition rather than reflecting an independent biological effect. Evidence on the relationship between alcohol exposure and hepatic steatosis among PLWH remains inconsistent. In the Copenhagen Co-morbidity Liver Study, which excluded individuals with excessive alcohol consumption, low-to-moderate alcohol intake was inversely associated with moderate-to-severe hepatic steatosis [44]. By contrast, alcohol exposure was associated with a higher likelihood of MASLD in the univariable analysis of our control group. Differences in population characteristics, the measurement and coding of alcohol exposure, and residual confounding may have contributed to this discrepancy.

The interaction between sex and HIV status observed in the present analysis warrants comment. In the control group, the direction of the sex association matched that reported in the general population and in meta-analyses of PLWH, with a higher prevalence of MASLD among men. Among PLWH, the direction was reversed. Recent studies in people with HIV are closer to the pattern we observed in controls than to the one we observed in PLWH: in a multicentre cohort of 1472 people with HIV, MASH with fibrosis was less frequent in women than in men (4.8% vs. 9.2%), and in a cross-sectional cohort of 964, female sex was independently protective against MASLD with clinically significant fibrosis (OR 0.43; 95% CI, 0.23–0.81) [45,46]. Sexual dimorphism in hepatic fat handling is biologically plausible, because sex chromosomes and sex hormones both influence hepatic lipid metabolism [47]. Because only 23 women were included in the PLWH group, the female stratum is imprecise, and this interaction should be regarded as exploratory and hypothesis-generating rather than established. The male stratum, comprising 227 of 250 PLWH, is adequately sized and gives a consistent result. Newly diagnosed, ART-naïve men living with HIV carried no excess burden of hepatic steatosis relative to HIV-negative men; indeed, their burden was lower. Baseline data cannot establish whether this reflects differences in body composition, nutritional status at diagnosis, or unmeasured behavioural factors. Prospective follow-up of the MASHIV-TR cohort, with more women, will be required to test whether the interaction is reproducible.

In our study, CD4 cell count, CD8 cell count, the CD4/CD8 ratio, and HIV RNA levels were not independently associated with CAP, LSM, MASLD, or the FAST score. HIV infection has been proposed to contribute to liver injury through chronic immune activation, persistent inflammation, and immunometabolic alterations [48]. In our baseline analyses, however, immunological and virological markers were not independently associated with liver outcomes. This finding is consistent with previous studies. In the Multicenter AIDS Cohort Study, neither nadir CD4 cell count nor detectable HIV RNA was associated with hepatic steatosis, whereas visceral adiposity and insulin resistance emerged as independent determinants [34]. Nevertheless, our baseline data cannot establish the potential effects of these markers on the long-term course of liver disease. Prospective follow-up within the MASHIV-TR study will allow us to evaluate how these relationships evolve after ART initiation. In cohorts followed after treatment initiation, CAP-defined steatosis rose from 23.1% before ART to 31.2% at one year, and new hepatic steatosis was detected in approximately one in ten participants within 48 weeks [49,50].

In this baseline analysis, noninvasive assessments using CAP, LSM, the FIB-4 index, and FAST did not indicate a greater burden of liver disease in newly diagnosed, ART-naïve PLWH than in HIV-negative controls. LSM remained comparable between the groups across all analyses. In contrast, differences in CAP, FAST, and FIB-4 were model-dependent and were not consistently confirmed in sensitivity analyses. These findings suggest that small between-group differences should not be interpreted on the basis of a single noninvasive measure. Current international guidelines similarly recommend the complementary use of serum-based scores and elastography for fibrosis risk assessment in MASLD, starting with the FIB-4 index and proceeding to elastography when indicated [20,21]. Taken together, these findings support the combined interpretation of noninvasive markers that capture different aspects of liver disease to characterise the baseline liver profile more comprehensively.

Several limitations should be acknowledged. Although MASHIV-TR is a prospective study, the present analysis was based exclusively on cross-sectional baseline data; therefore, causal relationships could not be established. Because liver biopsy was not performed, histological validation was unavailable, and liver health was assessed using noninvasive methods. As in all observational studies, residual confounding cannot be completely excluded. Missing data for some variables also reduced the sample size available for the corresponding analyses.

The marked difference in sex distribution between the groups was the principal source of potential confounding. It reflects the male-predominant epidemiology of newly diagnosed HIV infection in Türkiye rather than a sampling artefact. Matching the control group for sex would have improved internal comparability at the cost of external validity. Multivariable adjustment, overlap weighting with exact covariate balance, a male-only analysis, and direct sex-standardisation yielded concordant results. As noted above, the small number of women in the PLWH group limits the interaction between sex and HIV status, which should be regarded as exploratory.

Although the use of a single operator eliminated inter-observer variability, intra-observer reproducibility was not formally assessed, and no repeat-measurement substudy was undertaken. Examination date and study centre were not retained in the anonymised analysis dataset, so device drift over the study period and centre-level heterogeneity could not be examined. The operator was, however, a certified physician with more than 3000 prior examinations, making a learning-curve effect unlikely, and the distribution of the IQR-to-median ratio was narrow in both groups.

The control group was recruited by convenience sampling among outpatient attendees rather than from the general population. Although the low prevalences of diabetes (5.0%) and hypertension (2.5%), together with a MASLD prevalence well below that reported in Turkish clinic-based and at-risk populations, argue against enrichment for metabolic disease, selection bias cannot be excluded. The control group should therefore be interpreted as a contemporaneous clinic-based comparison group rather than as a representative population sample.

Chronic carriage of cytomegalovirus and Epstein–Barr virus was not assessed, and occupational exposure to hepatotoxic agents was not recorded. Latent herpesvirus infection is unlikely to have influenced CAP, which quantifies steatosis rather than hepatocellular inflammation, but unrecognised subclinical hepatic inflammation of any cause could in principle have affected liver stiffness measurement and the FAST score. Finally, because the present study reflects liver health at the time of HIV diagnosis, the potential effects of HIV-specific immunological and virological markers on long-term liver outcomes could not be evaluated.

## 4. Materials and Methods

### 4.1. Study Design and Participants

This study is the baseline analysis of a prospective, multicentre, observational cohort conducted between April 2024 and June 2025 in the Departments of Infectious Diseases and Clinical Microbiology at nine university hospitals in Türkiye. In this cohort, which was designed for long-term follow-up, metabolic status and liver health were evaluated before antiretroviral therapy (ART) was initiated in newly diagnosed people living with HIV (PLWH). The MASHIV-TR study is registered at ClinicalTrials.gov (NCT07737340).

A total of 411 participants were included in the study. Of these, 250 were newly diagnosed PLWH who had not yet initiated ART at the time of diagnosis (Group 1), and 161 were adult HIV-negative volunteers who constituted the control group (Group 2). The inclusion criteria for Group 1 were age ≥ 18 years, a new diagnosis of HIV infection, no previous exposure to ART, and voluntary provision of written informed consent. Group 2 comprised HIV-negative individuals aged ≥ 18 years who attended the outpatient clinics of the participating centres during the same period and voluntarily agreed to participate. The exclusion criteria for both groups were chronic hepatitis B or hepatitis C infection, known chronic liver disease, organ failure, haematologic or solid-organ malignancy, use of immunosuppressive therapy, pregnancy, and the presence of a severe systemic disease that could affect metabolic assessment. Alcohol consumption was assessed from participant self-report using standard drink units. Daily, weekly, or monthly consumption frequency was recorded, and all values were converted to grams of alcohol consumed per week, assuming that one standard drink contained approximately 14 g of pure alcohol. Individuals whose weekly alcohol consumption exceeded the thresholds proposed for metabolic dysfunction-associated steatotic liver disease with increased alcohol intake (MetALD) in the 2023 multisociety consensus statement and the 2024 EASL-EASD-EASO Clinical Practice Guidelines (>140 g/week for women and >210 g/week for men) were excluded from the study [21]. Controls were recruited by convenience sampling among general outpatient clinic attendees rather than by population-based or matched sampling, and no quota was applied for age or sex. Accompanying relatives and carers of patients were not enrolled, and individuals attending for the evaluation of suspected liver or metabolic disease were not eligible. All exclusion criteria listed above were applied identically to both groups. Chronic carriage of other potentially hepatotropic viruses, in particular cytomegalovirus and Epstein–Barr virus, was not used as a separate exclusion criterion. Latent infection with these viruses is near-universal among adults in Türkiye and therefore does not discriminate between the groups. Clinically active cytomegalovirus or Epstein–Barr virus hepatitis, by contrast, is an acute illness that the exclusion criteria for severe systemic disease and known liver disease would have captured. Occupational exposure to hepatotoxic agents was not systematically recorded.

Written informed consent was obtained from all participants before enrolment. The study protocol was approved by the Dicle University Faculty of Medicine Non-Interventional Clinical Research Ethics Committee (20 March 2024; decision no. 112), and the study was conducted in accordance with the principles of the Declaration of Helsinki, as revised in 2013.

A standardised data collection form was completed for all participants who met the inclusion criteria, had no exclusion criteria, and provided written informed consent. Demographic characteristics, anthropometric measurements, comorbidities, medication use, and lifestyle characteristics were recorded. The demographic and anthropometric assessment included age, sex, height, weight, body mass index (BMI), and waist and hip circumferences. BMI was calculated as body weight in kilograms divided by the square of height in metres (kg/m^2^). According to the World Health Organization classification, BMI was categorised as underweight (<18.5 kg/m^2^), normal weight (18.5–24.9 kg/m^2^), overweight (25.0–29.9 kg/m^2^), or obesity (≥30.0 kg/m^2^) [51]. Waist circumference was measured while the participant was standing, at the midpoint between the lower costal margin and the iliac crest, at the end of normal expiration; abdominal obesity was defined as a waist circumference of ≥94 cm in men and ≥80 cm in women [21]. Hip circumference was measured at the widest part of the gluteal region.

Hypertension, diabetes mellitus, coronary artery disease, other comorbidities, and the use of antihypertensive, antidiabetic, and statin medications were recorded on the basis of participant self-report, and this information was verified against electronic prescription records. Lifestyle assessment included smoking, alcohol and substance use, physical activity level, daily sleep duration, dietary habits (fast-food-based diet, high-protein diet, Mediterranean-style diet, multiple dietary patterns, vegan diet, and vegetarian diet), and supplement use. Menopausal status was also assessed in female participants. Weekly alcohol consumption was calculated in grams using standard drink units. One standard drink was considered to contain approximately 14 g of pure alcohol [21,52].

Laboratory assessments included white blood cell (WBC) count, platelet (PLT) count, ALT, AST, gamma-glutamyl transferase (GGT), alkaline phosphatase (ALP), lactate dehydrogenase (LDH), total and direct bilirubin, albumin, creatinine, urea, uric acid, phosphorus, alpha-fetoprotein (AFP), fasting plasma glucose, fasting insulin, haemoglobin A1c (HbA1c), total cholesterol, low-density lipoprotein cholesterol (LDL-C), high-density lipoprotein cholesterol (HDL-C), non-HDL cholesterol, triglycerides, C-reactive protein (CRP), erythrocyte sedimentation rate (ESR), D-dimer, and thyroid function tests. In the PLWH group, CD4+ T-lymphocyte count, CD8+ T-lymphocyte count, the CD4/CD8 ratio, and plasma HIV RNA levels were also recorded.

The FIB-4 score was calculated using age, AST, ALT, and platelet count. In accordance with current guideline recommendations, FIB-4 values < 1.3, 1.3–2.67, and >2.67 were considered indicative of low, indeterminate, and high risk of fibrosis, respectively. Hepatic steatosis and liver stiffness were assessed by vibration-controlled transient elastography (VCTE) using a FibroScan^®^ 530 device (Echosens, Paris, France). Hepatic steatosis was quantified by the controlled attenuation parameter (CAP) and liver stiffness by liver stiffness measurement (LSM). To standardise measurements across centres, all VCTE assessments were performed by the same trained operator. For this purpose, appointments were scheduled in advance with the participating centres, and the operator travelled to each centre with the FibroScan^®^ device to perform the measurements on site. All measurements were obtained after a fasting period of at least eight hours. Reliability criteria were prespecified as at least 10 valid measurements together with an interquartile range (IQR)-to-median ratio ≤ 0.30, and were verified in real time during each examination. When these technical quality criteria were not met, the examination was repeated within the same session whenever possible. Repetition was triggered exclusively by the technical quality indicators—the number of valid measurements and the IQR-to-median ratio—and never by the CAP or LSM value obtained. The median IQR-to-median ratio was 11% (Q1–Q3, 8–18%; range 2–29%) among PLWH and 16% (Q1–Q3, 11–21%; range 2–59%) among controls. Five control examinations nevertheless had a recorded IQR-to-median ratio of 59%, above the prespecified threshold; these were retained in the primary analysis and excluded in a sensitivity analysis. This operator was a certified physician who had performed more than 3000 prior VCTE examinations, so the study period did not fall within the operator’s learning curve. Median CAP and LSM values were used in the analyses.

Metabolic dysfunction-associated steatotic liver disease (MASLD) was defined according to the 2023 multisociety Delphi consensus as the presence of hepatic steatosis, indicated by CAP ≥ 248 dB/m, together with at least one of five cardiometabolic risk factors [52]. These risk factors were overweight or obesity (BMI ≥ 25 kg/m^2^) or abdominal obesity (waist circumference ≥ 94 cm in men and ≥80 cm in women), impaired glucose metabolism [fasting plasma glucose ≥ 100 mg/dL, 2-h oral glucose tolerance test glucose ≥ 140 mg/dL, haemoglobin A1c ≥ 5.7%, a diagnosis of type 2 diabetes mellitus, or use of antidiabetic therapy], blood pressure ≥ 130/85 mmHg or use of antihypertensive therapy, triglyceride levels ≥ 150 mg/dL or use of lipid-lowering therapy, and HDL-C levels < 40 mg/dL in men and <50 mg/dL in women or use of lipid-lowering therapy. LDL-C levels and insulin resistance were evaluated as metabolic variables distinct from the cardiometabolic criteria used to define MASLD. An LDL-C level ≥ 130 mg/dL was considered indicative of elevated LDL-C. Blood samples were collected after an overnight fast of at least eight hours. HOMA-IR was calculated using the formula [fasting insulin (μU/mL) × fasting glucose (mg/dL)/405], and HOMA-IR ≥ 2.5 was considered indicative of insulin resistance [21,53,54].

Because the study was observational and there was no clinical indication for liver biopsy in the participants, liver biopsy was not performed. The FibroScan-AST (FAST) score was calculated using LSM, CAP, and serum AST levels. In the primary analyses, the FAST score was evaluated as a continuous variable. In the secondary analyses, a cutoff of FAST ≥ 0.35 was used, as proposed in the original derivation and global validation study [24]. LSM was evaluated as a binary variable using a threshold of 8.0 kPa; in accordance with current guidelines, this threshold was considered suggestive of increased liver stiffness warranting further evaluation [55].

An a priori sample size calculation was performed during the planning phase of the prospective MASHIV-TR study using G*Power software (version 3.1.9.7) (Heinrich-Heine-Universität Düsseldorf, Düsseldorf, Germany). The calculation was based on data reported by Gawrieh et al. on the prevalence of steatotic liver disease among people living with HIV [16]. Assuming an effect size (Cohen’s f) of 0.10, a two-sided significance level (α) of 0.05, and a statistical power of 95%, the minimum required total sample size was calculated as 312 participants. The present baseline analysis included 411 participants, thereby exceeding the predefined sample size requirement.

### 4.2. Statistical Analysis

All analyses were restricted to baseline observations. Continuous variables were assessed for normality using the Shapiro–Wilk test separately within the PLWH and HIV-negative control groups. Variables compatible with normality in both groups are presented as mean ± standard deviation; otherwise, they are reported as median with interquartile range (Q1–Q3). Categorical variables are presented as number and percentage. Percentages were calculated using the available observations for each variable.

Baseline continuous variables were compared using Welch’s independent-samples *t*-test when normality assumptions were satisfied and the Mann–Whitney U test otherwise. The principal liver and metabolic outcomes were analysed nonparametrically. For these outcomes, the Hodges–Lehmann estimator and its 95% confidence interval were used to quantify the PLWH-minus-control location shift, and rank-biserial correlation was reported as a nonparametric effect-size measure. Categorical variables were compared using Pearson’s chi-squared test or Fisher’s exact test, as appropriate. Unadjusted odds ratios and exact 95% confidence intervals for binary liver outcomes were obtained using Fisher’s exact test. Absolute standardised mean differences were additionally reported to quantify between-group imbalance independently of sample size.

Adjusted PLWH–control comparisons were performed using two prespecified models. Model 1 included age and sex, whereas Model 2 additionally included BMI. Continuous liver outcomes, including CAP, LSM, FAST and FIB-4, were analysed using median regression with τ = 0.50 and were reported as adjusted median differences with 95% confidence intervals. As a sensitivity analysis, ordinary linear regression with heteroscedasticity-consistent HC3 standard errors was used to estimate adjusted mean differences. Binary outcomes were analysed using mean bias-reduced logistic regression to reduce small-sample and separation bias, and the results were reported as adjusted odds ratios with 95% confidence intervals.

Propensity-score overlap weighting was conducted as a supporting analysis of the baseline PLWH–control comparisons. The propensity-score model included age, sex, body mass index, smoking, alcohol use, comorbidity, physical activity, sleep duration, lifestyle pattern, dietary pattern, educational status and triglyceride level. Overlap weights were calculated as 1−propensity score for PLWH and as the propensity score for controls. Propensity scores were constrained to the range 0.001–0.999. Missing continuous covariates in the propensity-score model were replaced by the corresponding sample median. Missing categorical values were retained as a separate category, and sparse categories were combined when necessary. Covariate balance was evaluated using absolute standardised mean differences, with a value < 0.10 taken to indicate adequate balance. Effective sample sizes were also calculated. Weighted linear and quasibinomial regression models were used for continuous and binary outcomes, respectively.

Because of the marked between-group difference in sex distribution, four additional analyses were specified. First, covariate balance before and after overlap weighting was reported, together with the effective sample size in each group. Second, the primary comparisons were repeated in a male-only subgroup (227 PLWH and 92 controls). Third, the prevalence of each binary outcome in the control group was directly standardised to the sex distribution of the PLWH group. Fourth, a multiplicative interaction between HIV status and sex was tested.

Within the PLWH group, associations of baseline clinical, metabolic, lifestyle and HIV-related factors with CAP, LSM, FAST and FIB-4 were initially examined. Spearman’s rank correlation was used for continuous predictors, the Mann–Whitney U test for two-category predictors and the Kruskal–Wallis test for predictors with more than two categories. Univariable associations with MASLD were evaluated using bias-reduced logistic regression. Outcome-specific multivariable analyses were subsequently performed using median regression for continuous liver outcomes and bias-reduced logistic regression for binary outcomes. Metabolic and HIV/lifestyle models were evaluated separately to limit overfitting and collinearity. Continuous predictors were rescaled to clinically interpretable increments where appropriate. Variables that were mathematical components of an outcome were excluded from the corresponding predictor model to avoid circular associations; therefore, CAP, LSM and AST were not entered as predictors of FAST, and age, AST, ALT and platelet count were not interpreted as independent determinants of FIB-4.

As an exploratory secondary analysis, factors associated with MASLD were also evaluated within the HIV-negative control group. Univariable associations were examined using bias-reduced logistic regression, with Benjamini–Hochberg false-discovery-rate correction applied across the screened predictors. Given the number of MASLD events, a parsimonious metabolic model was constructed, including body mass index per 5 kg/m^2^ increase, HOMA-IR per 1-unit increase, triglyceride level per 50 mg/dL increase and HDL-cholesterol level per 10 mg/dL increase. Variance inflation factors were examined to assess multicollinearity. As sensitivity analyses, an alternative model including age, sex, triglyceride and HDL-cholesterol was evaluated, and the primary metabolic model was repeated using the originally recorded MASLD classification.

Because triglyceride and HDL-cholesterol levels are components of the cardiometabolic criteria used to define MASLD, three analyses were performed to address definitional circularity. First, the number of participants with hepatic steatosis (CAP ≥ 248 dB/m) who also met at least one cardiometabolic criterion was determined to establish whether the cardiometabolic requirement was binding. Second, the proportion of steatotic participants who met that requirement exclusively through the triglyceride and/or HDL-cholesterol criterion was quantified. Third, MASLD was redefined using only the non-lipid cardiometabolic criteria—overweight or obesity, abdominal obesity and impaired glucose metabolism—and the association between triglyceride level and this non-lipid definition was re-examined in both groups. Agreement between the recalculated MASLD classification and the classification originally recorded at the participating centres was also assessed.

Analyses were conducted using variable-specific complete cases, except for the prespecified handling of missing propensity-score covariates described above; no missing outcome values were imputed. All tests were two-sided, and *p* < 0.05 was considered statistically significant. Benjamini–Hochberg false-discovery-rate correction was applied to exploratory factor-screening analyses in the PLWH and HIV-negative control groups, and to within-model multiple comparisons. Analyses were performed using R version 4.4.2 (R Foundation for Statistical Computing, Vienna, Austria).

During the revision of this work, Claude (Anthropic, Claude Opus 5) was used to assist with the supporting analyses added during revision—the four sex-related analyses and the three analyses addressing definitional circularity described above—and to generate Figure 1, Figure 2, Figure 3, Figure 4, Figure 5, Figure 6 and Figure 7 from the study results. The statistical analyses were carried out according to an analysis plan specified in advance by the authors; the tool was not used to define the analysis plan or to perform the primary statistical analyses, which were conducted by the authors in R version 4.4.2. All AI-assisted output was checked by the authors against the source data and the analysis output before it was included in the manuscript. The tool was not used for study design, for data collection, or for the generation, modification, imputation or any other processing of the study data.

## 5. Conclusions

The prevalence of MASLD among newly diagnosed, ART-naïve PLWH did not differ significantly from that among contemporaneously recruited HIV-negative outpatient controls. However, this overall comparison averaged opposite sex-specific patterns: among men, MASLD was significantly less frequent in PLWH. Triglyceride levels were independently associated with both CAP and MASLD among PLWH and showed a similar independent association with MASLD among HIV-negative controls. In contrast, HIV-specific immunological and virological markers were not independently associated with the liver outcomes evaluated. These findings suggest that, during the pre-ART period, MASLD may be more closely associated with metabolic processes than with HIV-specific virological or immunological factors. Continued prospective follow-up of the MASHIV-TR study will allow changes in metabolic and hepatic markers to be evaluated after ART initiation.

## Figures and Tables

**Figure 1:**
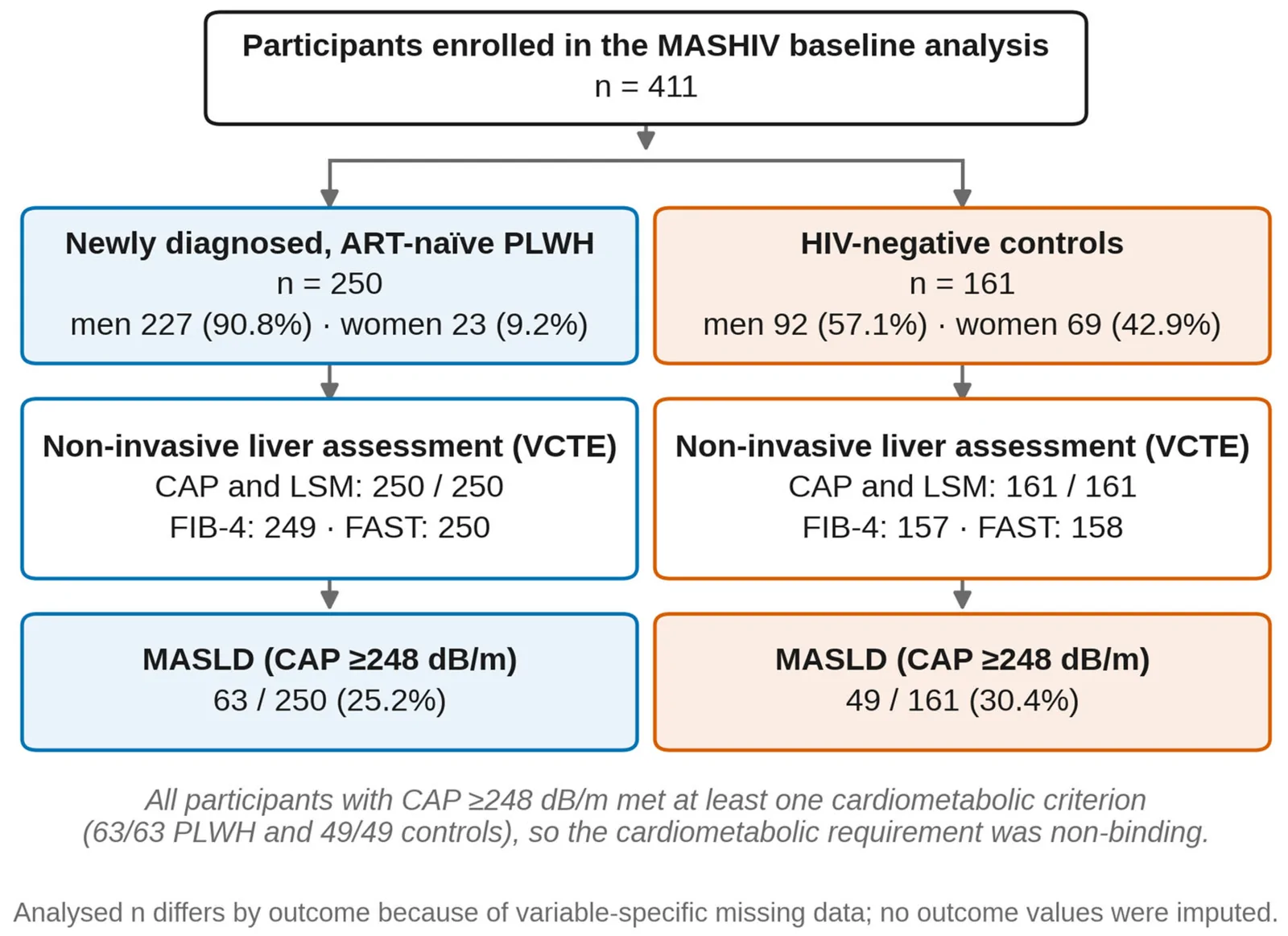
Participant flow and analysed sample. Of 411 participants, 250 were newly diagnosed, ART-naïve PLWH and 161 were HIV-negative controls. The analysed *n* differs between outcomes because variable-specific complete cases were used and no outcome values were imputed. Every participant with a controlled attenuation parameter (CAP) ≥ 248 dB/m also met at least one cardiometabolic criterion, so the cardiometabolic requirement of the MASLD definition was non-binding in this cohort. ART, antiretroviral therapy; FAST, FibroScan–AST score; FIB-4, Fibrosis-4 index; LSM, liver stiffness measurement; VCTE, vibration-controlled transient elastography.

**Figure 2:**
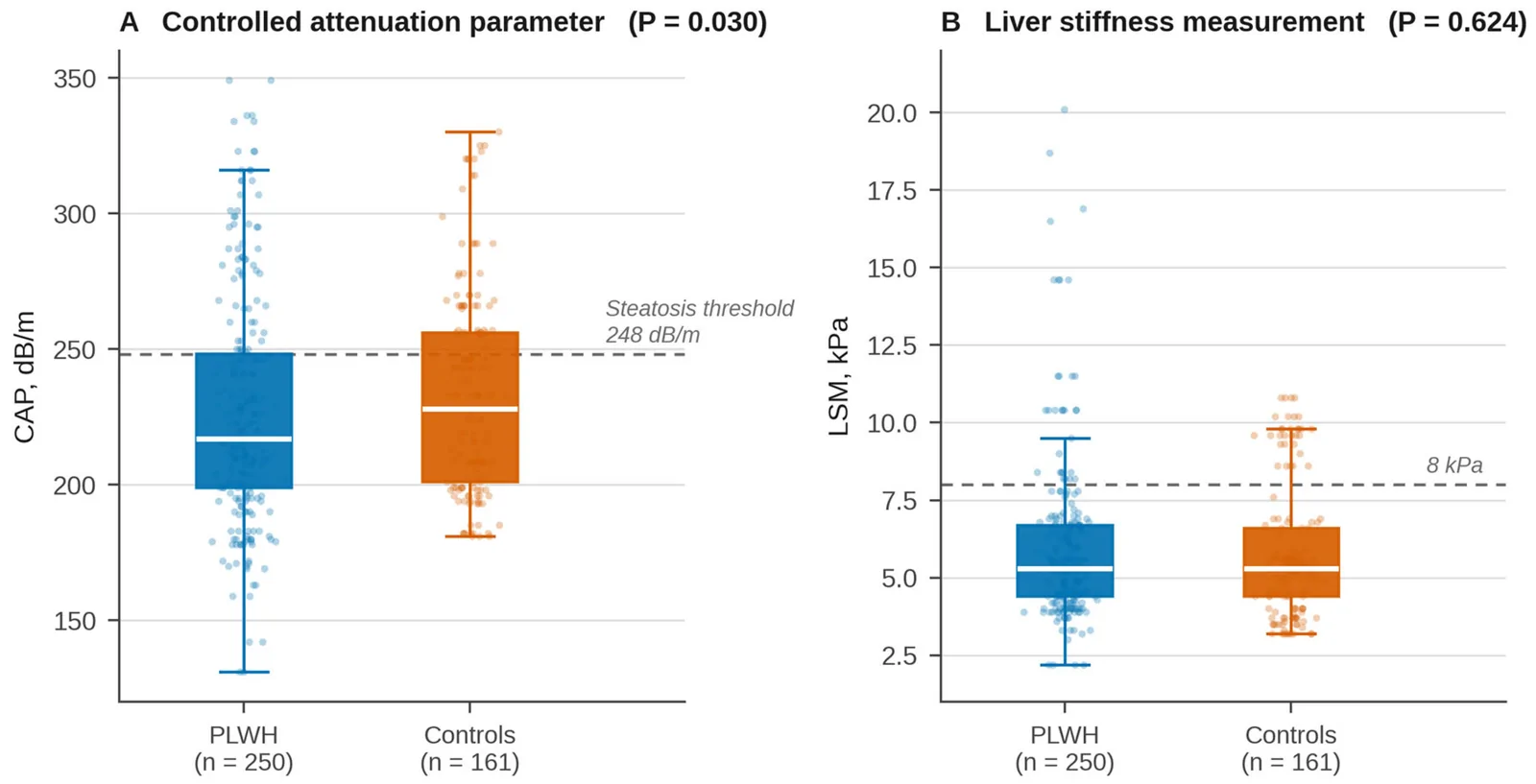
Distribution of CAP (**A**) and LSM (**B**) by HIV status. Boxes show the median and interquartile range with whiskers extending to 1.5 times the interquartile range; individual participants are overlaid. Dashed lines mark the steatosis threshold of 248 dB/m and the liver stiffness threshold of 8 kPa. *p* values are from the Mann–Whitney U test.

**Figure 3:**
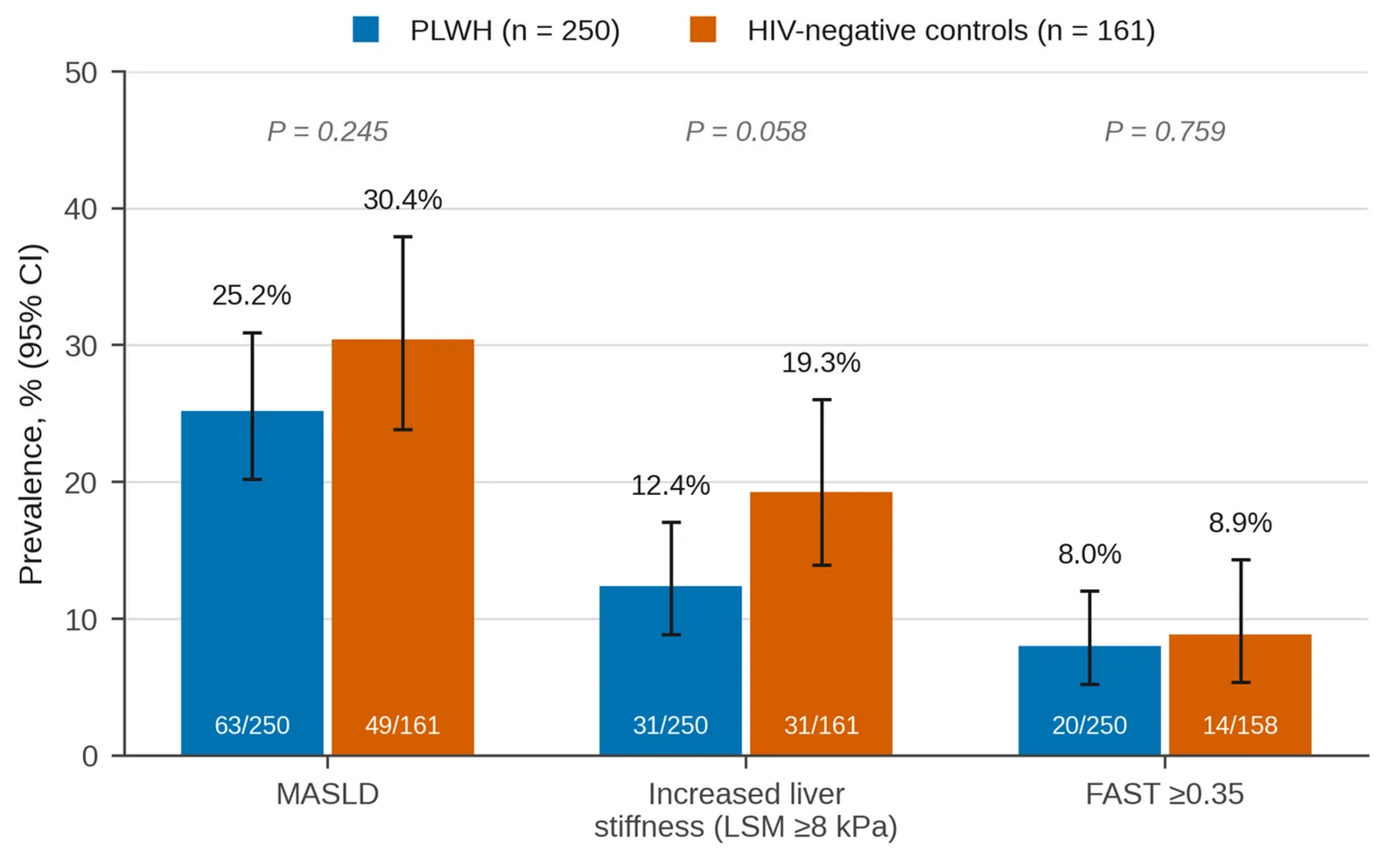
Prevalence of binary liver outcomes in PLWH and HIV-negative controls. Bars show the prevalence of MASLD (CAP ≥ 248 dB/m), increased liver stiffness (LSM ≥ 8 kPa) and a FAST score ≥ 0.35; error bars are Wilson 95% confidence intervals and the numerators and denominators are given inside each bar. *p* values are from Fisher’s exact test. FAST-based analyses included 158 controls because serum AST was unavailable in three participants.

**Figure 4:**
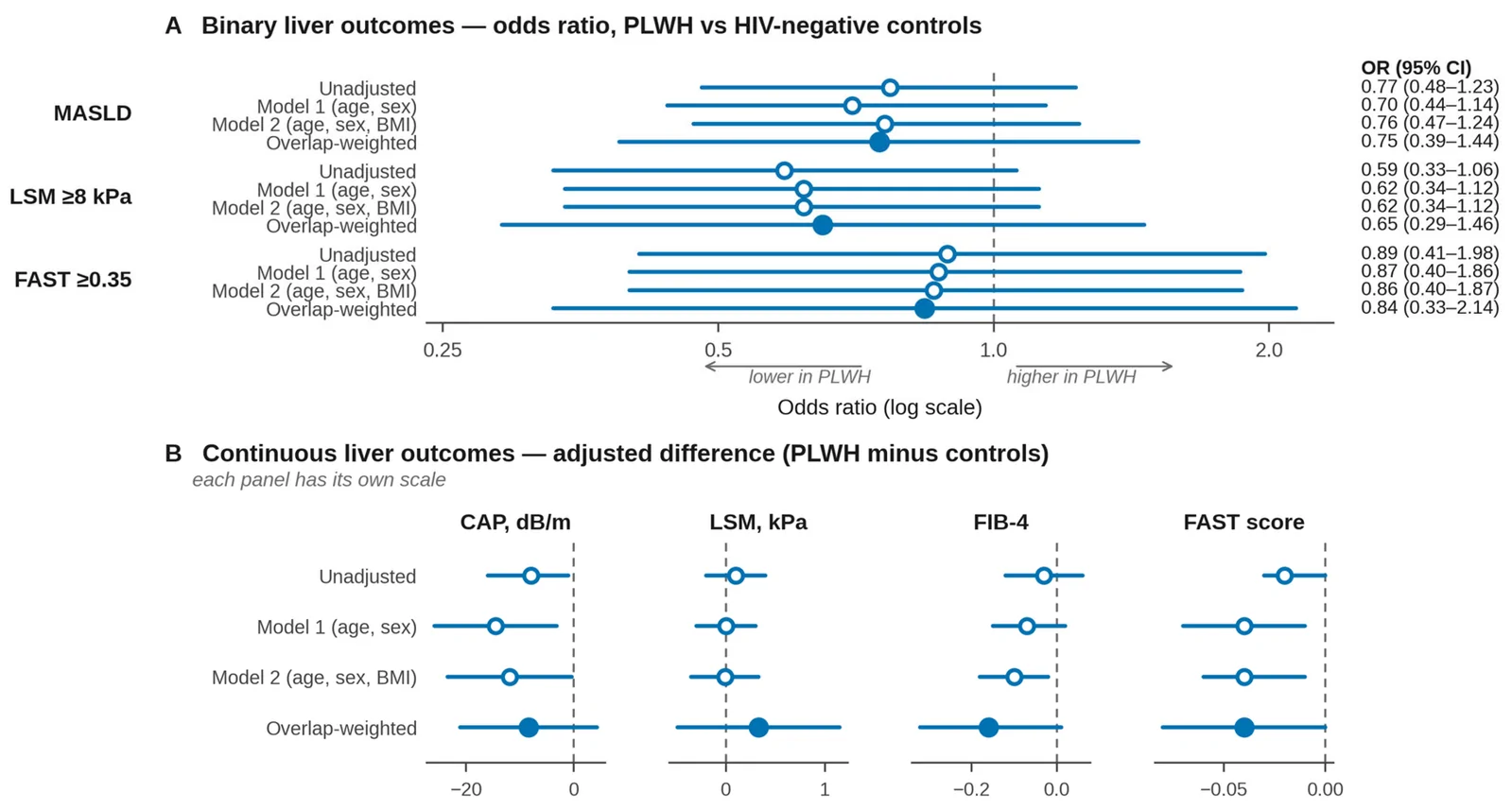
Association between HIV status and liver outcomes across adjustment strategies. (**A**) Odds ratios for binary outcomes, PLWH versus HIV-negative controls, on a logarithmic scale; an odds ratio below 1.0 indicates a lower prevalence among PLWH. (**B**) Adjusted differences for continuous outcomes, PLWH minus controls; each panel has its own scale because the outcomes are measured in different units. Model 1 was adjusted for age and sex and Model 2 additionally for body mass index. Filled circles denote the overlap-weighted estimates, in which the absolute standardised mean difference of every covariate, including sex, was reduced to below 0.001. Horizontal lines are 95% confidence intervals.

**Figure 5:**
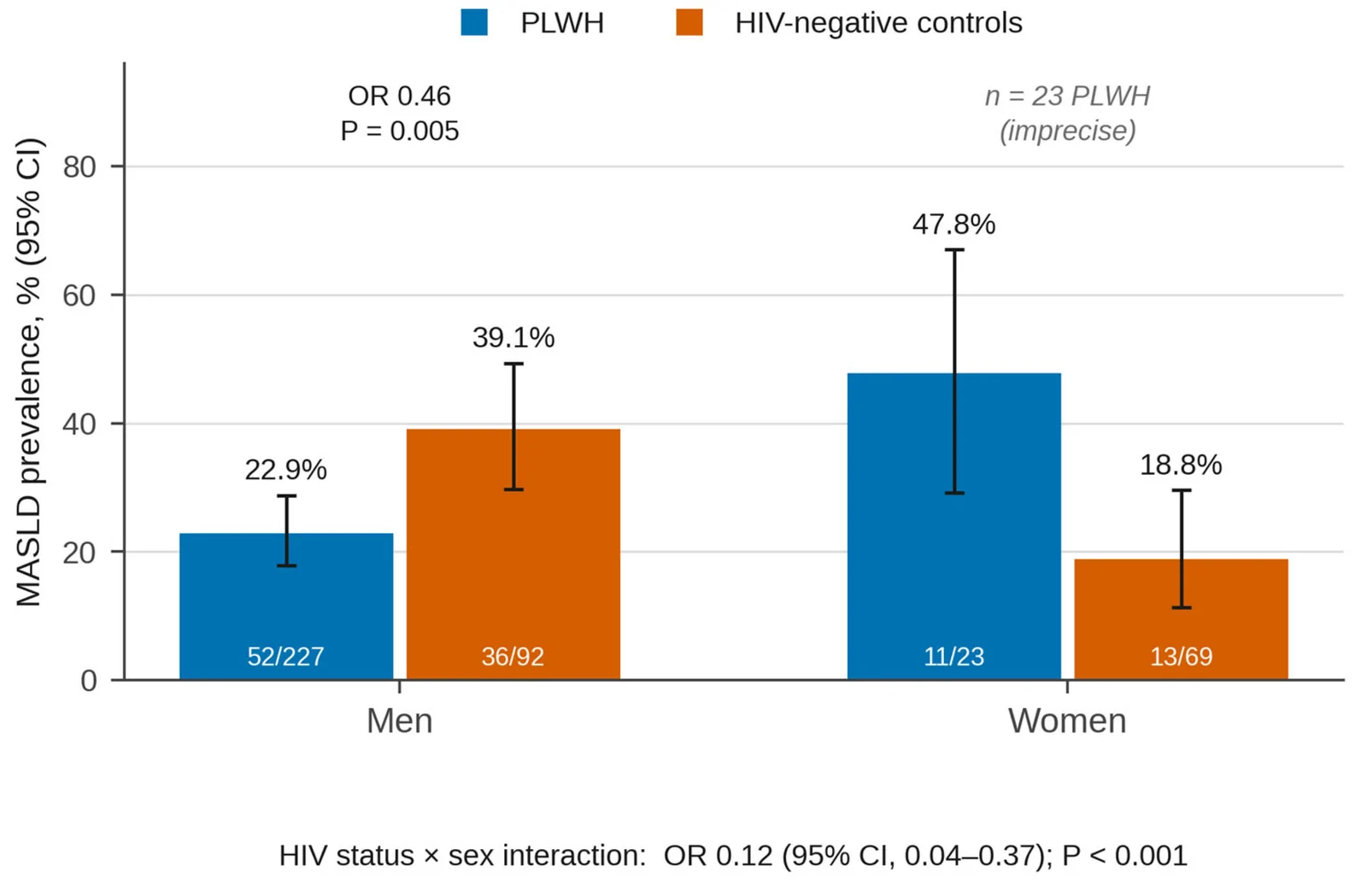
MASLD prevalence by HIV status and sex. Error bars are Wilson 95% confidence intervals; numerators and denominators are given inside each bar. Among men, MASLD was less frequent in PLWH than in controls. The estimate among women is based on 23 PLWH, is imprecise, and should be regarded as exploratory. The interaction between HIV status and sex was estimated in a logistic model including HIV status, sex and their product term.

**Figure 6:**
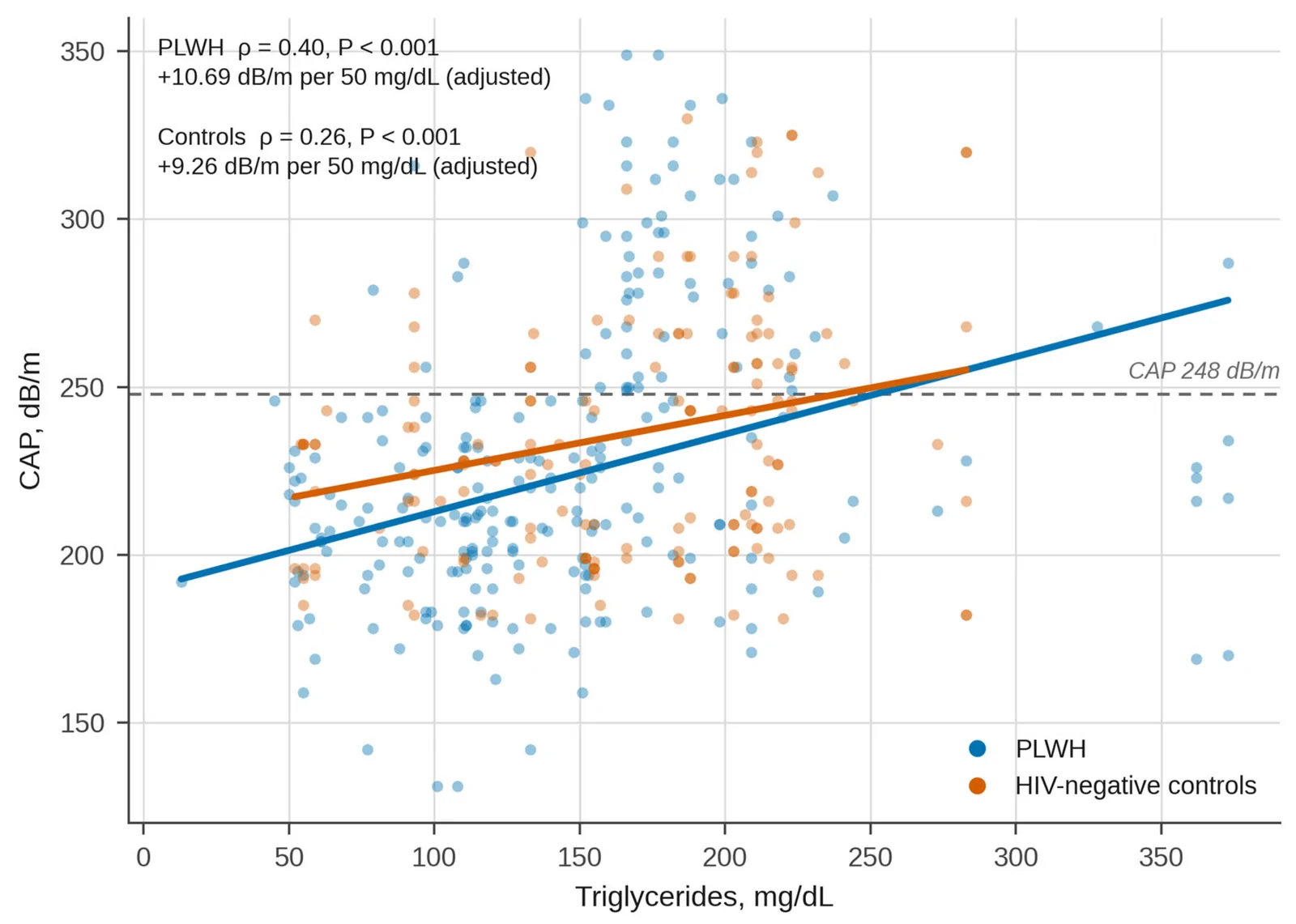
Triglyceride level and CAP in PLWH and HIV-negative controls. Each point is one participant; lines are median regression fits. The dashed line marks the steatosis threshold of 248 dB/m. Adjusted estimates are from median regression models including age, sex, body mass index, HOMA-IR and triglyceride level. The association was of similar magnitude in both groups, indicating that it is neither specific to HIV infection nor an artefact of the MASLD definition, since CAP incorporates no cardiometabolic criterion.

**Figure 7:**
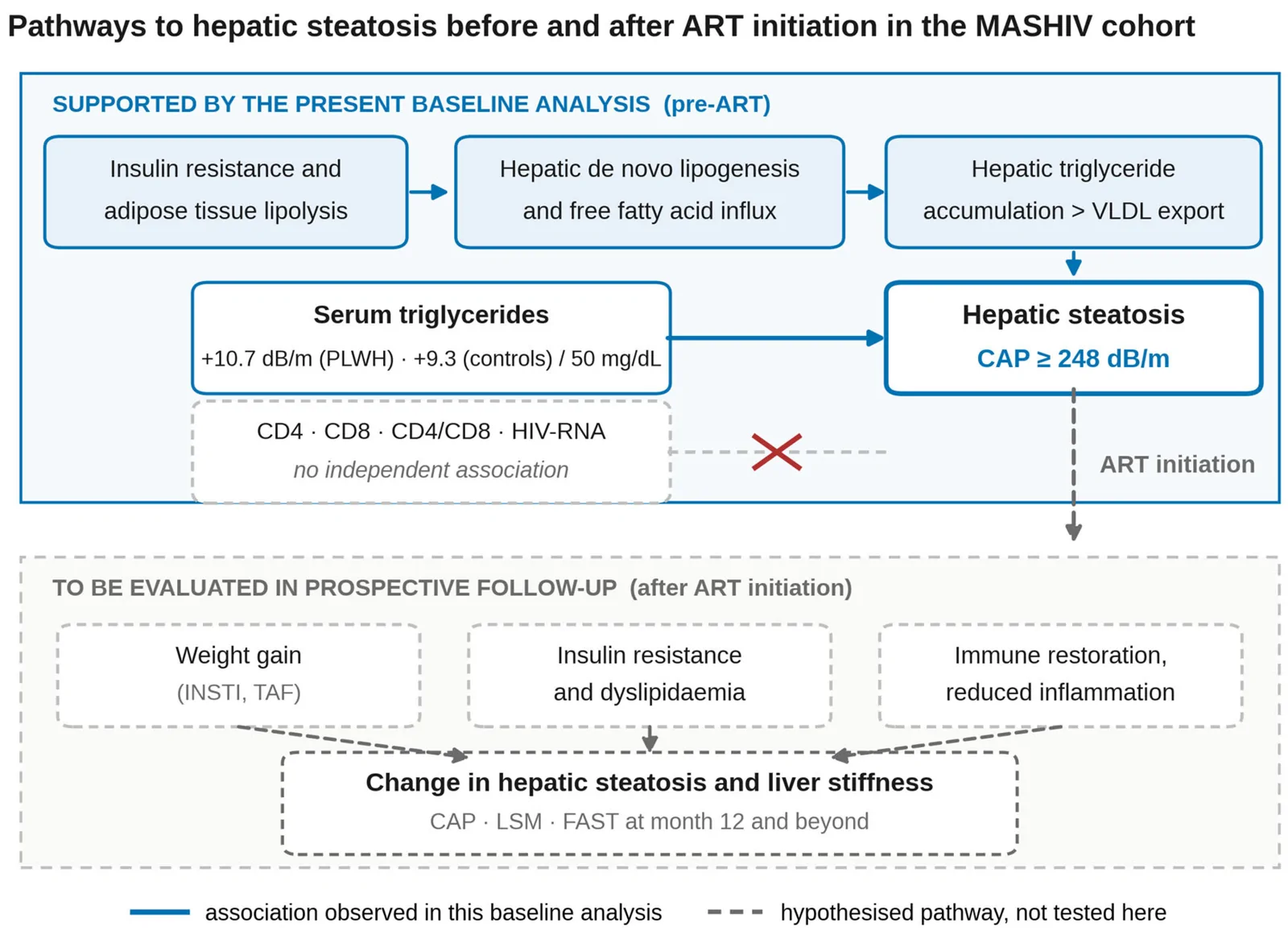
Pathways to hepatic steatosis before and after ART initiation. Solid blue elements denote associations observed in the present baseline analysis: the metabolic route from insulin resistance and adipose tissue lipolysis to hepatic triglyceride accumulation and steatosis, and the association between serum triglyceride level and CAP in both groups. The crossed dashed arrow indicates that CD4 and CD8 cell counts, the CD4/CD8 ratio and HIV RNA level were not independently associated with any liver outcome. The cross (×) denotes the absence of an independent association. Dashed grey elements denote pathways that were not tested in this cross-sectional baseline analysis and that will be evaluated during prospective follow-up after ART initiation. INSTI, integrase strand transfer inhibitor; TAF, tenofovir alafenamide.

**Table 1:** Baseline demographic, clinical, and laboratory characteristics of the study population.

Characteristic	PLWH (*n* = 250)	HIV-Negative Controls (*n* = 161)	*p* Value ^a^	SMD ^b^	Overlap-Weighted SMD
**Demographic characteristics**					
Age, years	34.00 (28.00–44.00)	30.00 (27.00–43.00)	**0.023**	0.22	<0.001
Male sex	227 (90.8%)	92 (57.1%)	**<0.001**	0.83	<0.001
Education			**<0.001**	0.39	<0.001
High school	81 (32.7%)	58 (36.0%)			
Illiterate	1 (0.4%)	0 (0.0%)			
Middle school	43 (17.3%)	13 (8.1%)			
Primary school	37 (14.9%)	6 (3.7%)			
University	86 (34.7%)	84 (52.2%)			
**Anthropometric profile**					
Body weight, kg	76.00 (65.00–84.00)	79.00 (66.00–88.00)	0.079	0.28	—
BMI, kg/m^2^	24.24 (22.21–27.64)	26.23 (23.66–28.09)	**<0.001**	0.43	<0.001
Waist circumference, cm	87.00 (80.00–96.00)	89.00 (81.00–97.00)	0.188	0.08	—
Hip circumference, cm	98.00 (93.00–104.00)	99.00 (95.00–106.00)	**0.042**	0.20	—
Waist-to-hip ratio	0.87 (0.85–0.94)	0.87 (0.83–0.95)	0.830	0.05	—
**Metabolic comorbidities and medication use**					
Diabetes mellitus	18 (7.2%)	8 (5.0%)	0.364	0.09	—
Hypertension	16 (6.4%)	4 (2.5%)	0.072	0.19	—
Coronary artery disease	16 (6.4%)	0 (0.0%)	**0.001**	0.37	—
Any comorbidity	79 (31.6%)	25 (15.5%)	**<0.001**	0.39	<0.001
**Lifestyle factors**					
Current smoking	138 (55.2%)	65 (40.4%)	**0.003**	0.30	<0.001
Alcohol use	86 (34.5%)	42 (26.1%)	0.071	0.18	<0.001
Physical activity			0.083	0.24	<0.001
Exercise at least once per week	88 (35.2%)	57 (35.4%)			
No regular exercise	143 (57.2%)	100 (62.1%)			
Regular daily exercise	19 (7.6%)	4 (2.5%)			
Lifestyle pattern			<0.001	0.69	<0.001
Intensive work schedule	33 (13.2%)	68 (42.2%)			
Night-oriented lifestyle	11 (4.4%)	0 (0.0%)			
Regular lifestyle	119 (47.6%)	44 (27.3%)			
Sedentary lifestyle	87 (34.8%)	49 (30.4%)			
**Glucose metabolism and insulin resistance**					
FPG, mg/dL	87.00 (80.00–98.00)	82.00 (77.00–91.00)	**<0.001**	0.44	—
HbA1c, %	5.60 (5.40–5.80)	5.60 (5.40–5.90)	0.272	0.04	—
Fasting insulin, µIU/mL	6.80 (4.15–11.60)	5.70 (3.94–10.00)	**0.038**	0.14	—
HOMA-IR	1.49 (0.91–2.60)	1.17 (0.75–1.98)	**0.002**	0.25	—
**Lipid profile**					
TG, mg/dL	140.00 (108.00–177.00)	177.00 (121.00–211.00)	**<0.001**	0.31	<0.001
TC, mg/dL	161.00 (134.00–185.00)	146.00 (125.75–174.00)	**0.018**	0.13	—
HDL-C, mg/dL	37.00 (32.00–42.00)	35.00 (30.75–42.00)	0.057	0.18	—
LDL-C, mg/dL	108.00 (85.00–125.25)	104.50 (86.75–121.00)	0.555	0.03	—
Non-HDL-C, mg/dL	121.00 (98.00–145.75)	103.00 (93.75–138.00)	**0.012**	0.10	—
**Haematological and inflammatory markers**					
WBC, ×10^3^/µL	6.88 (5.50–8.10)	6.00 (5.20–7.95)	**0.025**	0.26	—
Platelets, ×10^3^/µL	226.00 (195.00–263.00)	213.00 (181.00–226.00)	**<0.001**	0.51	—
**Liver and renal biochemistry**					
ALT, U/L	23.00 (16.00–31.00)	26.00 (19.75–46.00)	**0.018**	0.20	—
AST, U/L	23.00 (20.00–31.00)	24.00 (20.00–36.00)	0.085	0.18	—
GGT, U/L	23.00 (17.00–29.00)	22.00 (18.00–29.75)	0.986	0.05	—
Albumin, g/dL	4.37 (4.13–4.75)	4.47 (4.16–4.76)	0.213	0.15	—
Creatinine, mg/dL	0.81 (0.70–0.98)	0.80 (0.60–0.90)	**<0.001**	0.42	—
**HIV-related characteristics (PLWH only)**					
CD4 count, cells/µL	371.50 (240.00–574.00)	—	—	—	—
CD8 count, cells/µL	962.00 (709.25–1400.00)	—	—	—	—
CD4/CD8 ratio	0.39 (0.22–0.60)	—	—	—	—
HIV RNA, copies/mL	93,700.00 (32,500.00–326,900.00)	—	—	—	—
HIV RNA, log10 copies/mL	4.97 (4.51–5.51)	—	—	—	—

**Notes.** Continuous variables are presented as median (Q1–Q3), and categorical variables as *n* (%). ^a^ *p* values compare PLWH with HIV-negative controls and were obtained using Welch’s *t* test, the Mann–Whitney U test, Pearson’s chi-squared test, or Fisher’s exact test, as appropriate. ^b^ SMD is the absolute standardised mean difference; values of approximately 0.10, 0.20 and 0.50 indicate small, moderate and large imbalance. Overlap-weighted SMD is the corresponding value after propensity-score overlap weighting, reported for covariates included in that model; a dash marks covariates not included. Variable-specific complete cases were used. The available PLWH/control observations ranged from 232/145 (hip circumference and waist-to-hip ratio) to 250/161; HIV-related variables were available for all 250 PLWH. Bold *p* values indicate *p* < 0.05; all tests were two-sided. Abbreviations: ALT, alanine aminotransferase; AST, aspartate aminotransferase; BMI, body mass index; FPG, fasting plasma glucose; GGT, gamma-glutamyl transferase; HbA1c, glycated haemoglobin; HDL-C, high-density lipoprotein cholesterol; HIV, human immunodeficiency virus; HOMA-IR, Homeostatic Model Assessment of Insulin Resistance; LDL-C, low-density lipoprotein cholesterol; PLWH, people living with HIV; SMD, standardised mean difference; TC, total cholesterol; TG, triglycerides; WBC, white blood cell count.

**Table 2:** Baseline liver assessment and liver-related outcomes.

Outcome	PLWH (*n* = 250)	HIV-Negative Controls (*n* = 161)	*p* Value ^a^	SMD ^b^
Continuous liver measures				
CAP, dB/m	217.00 (199.00–248.25)	228.00 (201.00–256.00)	**0.030**	0.18
LSM, kPa	5.30 (4.40–6.70)	5.30 (4.40–6.60)	0.624	0.06
FIB-4	0.78 (0.54–1.19)	0.82 (0.59–1.17)	0.488	0.09
FAST score	0.10 (0.06–0.19)	0.13 (0.07–0.22)	0.065	0.15
Binary liver outcomes				
CAP-defined steatosis (≥248 dB/m)	63 (25.2%)	49 (30.4%)	0.245	0.12
LSM ≥ 8 kPa	31 (12.4%)	31 (19.3%)	0.058	0.19
MASLD	63 (25.2%)	49 (30.4%)	0.245	0.12
FAST ≥ 0.35	20 (8.0%)	14 (8.9%)	0.759	0.03

Notes. Continuous variables are presented as median (Q1–Q3), and binary outcomes as *n* (%). ^a^ Continuous outcomes were compared using the Mann–Whitney U test. Binary outcomes were compared using Pearson’s chi-squared test or Fisher’s exact test, as appropriate. CAP-defined steatosis, CAP ≥ 248 dB/m; MASLD, CAP ≥ 248 dB/m with at least one cardiometabolic risk factor; elevated liver stiffness, LSM ≥ 8 kPa. FAST ≥ 0.35 is a noninvasive phenotype and should not be read as histologically confirmed MASH. Every participant with CAP ≥ 248 dB/m met at least one cardiometabolic criterion, so the two prevalences are identical. ^b^ SMD is the absolute standardised mean difference and is presented as a measure of between-group imbalance; values of approximately 0.10, 0.20, and 0.50 indicate small, moderate, and large imbalance, respectively. Available PLWH/control observations were: CAP, dB/m, 250/161; LSM, kPa, 250/161; FIB-4, 249/157; FAST score, 250/158; CAP-defined steatosis (≥248 dB/m), 250/161; LSM ≥ 8 kPa, 250/161; MASLD, 250/161; FAST ≥ 0.35, 250/158. Analyses used variable-specific complete cases without missing-value imputation. Bold *p* values indicate *p* < 0.05; all tests were two-sided. Abbreviations: FAST, FibroScan–AST score; CAP, controlled attenuation parameter; FIB-4, Fibrosis-4 index; HIV, human immunodeficiency virus; LSM, liver stiffness measurement; MASLD, metabolic dysfunction-associated steatotic liver disease; MASH, metabolic dysfunction-associated steatohepatitis; PLWH, people living with HIV; SMD, standardised mean difference.

**Table 3:** Crude and adjusted comparisons of liver outcomes between PLWH and HIV-negative controls.

Outcome	Unadjusted Estimate (95% CI); *p* Value	Model 1 Estimate (95% CI); *p* Value	Model 2 Estimate (95% CI); *p* Value	Sensitivity Estimate (95% CI); *p* Value
**Panel A. Continuous liver outcomes**				
CAP, dB/m	−8.00 (−16.00 to −1.00); 0.030	−14.50 (−25.82 to −3.18); 0.012	−11.91 (−23.45 to −0.37); 0.044	−6.07 (−15.58 to 3.44); 0.211
LSM, kPa	0.10 (−0.20 to 0.40); 0.624	−0.00 (−0.30 to 0.30); 1.000	−0.01 (−0.35 to 0.33); 0.956	0.14 (−0.35 to 0.63); 0.578
FIB-4	−0.03 (−0.12 to 0.06); 0.488	−0.07 (−0.15 to 0.02); 0.121	−0.10 (−0.18 to −0.02); 0.017	−0.12 (−0.23 to −0.00); 0.048
FAST score	−0.02 (−0.03 to 0.00); 0.065	−0.04 (−0.07 to −0.01); 0.003	−0.04 (−0.06 to −0.01); 0.007	−0.02 (−0.05 to 0.00); 0.095
**Panel B. Binary liver outcomes**				
MASLD	0.77 (0.48 to 1.23); 0.258	0.70 (0.44 to 1.14); 0.150	0.76 (0.47 to 1.24); 0.277	—
FAST ≥ 0.35	0.89 (0.41 to 1.98); 0.854	0.87 (0.40 to 1.86); 0.712	0.86 (0.40 to 1.87); 0.709	—
CAP-defined steatosis (≥248 dB/m)	0.77 (0.48 to 1.23); 0.258	0.70 (0.44 to 1.14); 0.150	0.76 (0.47 to 1.24); 0.277	—
LSM ≥ 8 kPa	0.59 (0.33 to 1.06); 0.067	0.62 (0.34 to 1.12); 0.111	0.62 (0.34 to 1.12); 0.112	—

**Notes.** Continuous estimates are PLWH-minus-control differences; binary estimates are odds ratios for PLWH versus HIV-negative controls (reference group). Model 1 was adjusted for age and sex. Model 2 was adjusted for age, sex, and BMI. Continuous primary analyses used median regression; sensitivity analyses used linear regression with HC3 standard errors. Binary outcomes used mean bias-reduced logistic regression. Continuous outcomes are Hodges–Lehmann location shifts from Mann–Whitney U tests; binary outcomes are odds ratios with exact 95% confidence intervals from Fisher exact tests. Analysed *n* was 250/161 for CAP, LSM, MASLD, CAP-defined steatosis and LSM ≥8 kPa, 249/157 for FIB-4 and 250/158 for FAST; adjusted totals were 405–411 depending on the outcome and model. All tests were two-sided; *p* < 0.05 was considered statistically significant. No multiplicity adjustment was applied to these prespecified between-group comparisons. Abbreviations: BMI, body mass index; CAP, controlled attenuation parameter; CI, confidence interval; FIB-4, Fibrosis-4 index; HIV, human immunodeficiency virus; LSM, liver stiffness measurement; MASLD, metabolic dysfunction-associated steatotic liver disease; PLWH, people living with HIV.

**Table 4:** Factors associated with liver outcomes among people living with HIV.

Outcome	Factor	Effect Estimate	*p* Value	FDR-Adjusted *p*
**Panel A. FDR-significant nonparametric screening associations**				
CAP, dB/m	BMI, kg/m^2^	0.19	0.003	0.017
CAP, dB/m	FPG, mg/dL	0.24	<0.001	0.001
CAP, dB/m	GGT, U/L	0.17	0.008	0.039
CAP, dB/m	HDL-C, mg/dL	−0.28	<0.001	<0.001
CAP, dB/m	HDL/LDL-C ratio	−0.41	<0.001	<0.001
CAP, dB/m	LDL-C, mg/dL	0.28	<0.001	<0.001
CAP, dB/m	TG, mg/dL	0.40	<0.001	<0.001
FIB-4	HbA1c, %	0.23	<0.001	0.016
**Panel B. FDR-significant univariable associations with MASLD**				
MASLD	ALT, U/L	1.02 (1.01 to 1.04)	0.002	0.011
MASLD	HDL-C, mg/dL	0.90 (0.86 to 0.94)	<0.001	<0.001
MASLD	HDL/LDL-C ratio	0.00 (0.00 to 0.04)	<0.001	<0.001
MASLD	LDL-C, mg/dL	1.02 (1.01 to 1.03)	<0.001	<0.001
MASLD	TG, mg/dL	1.01 (1.01 to 1.02)	<0.001	<0.001
**Panel C. Multivariable associations**				
CAP, dB/m	Triglycerides, per 50 mg/dL	10.69 (5.51 to 15.88)	<0.001	<0.001
CAP, dB/m	BMI, per 5 kg/m^2^	6.99 (0.42 to 13.57)	0.038	0.343
LSM, kPa	BMI, per 5 kg/m^2^	0.32 (0.02 to 0.61)	0.036	0.323
MASLD	Triglycerides, per 50 mg/dL	1.70 (1.32 to 2.18)	<0.001	<0.001
MASLD	BMI, per 5 kg/m^2^	1.47 (1.05 to 2.06)	0.023	0.140
MASLD	Male sex	0.37 (0.15 to 0.93)	0.035	0.140

**Notes.** Continuous predictors were assessed with Spearman rank correlation. Univariable MASLD associations were estimated using mean bias-reduced logistic regression. Only screening findings with Benjamini–Hochberg FDR-adjusted *p* < 0.05 are shown. Model A evaluated prespecified metabolic factors; Model B evaluated HIV-related and lifestyle factors. Adjusted continuous-outcome estimates are median differences; adjusted MASLD estimates are odds ratios. Multivariable rows with nominal *p* < 0.05 are shown together with within-model FDR-adjusted *p* values. Association does not imply causation. Triglycerides are also included among the cardiometabolic criteria used to define MASLD; associations with MASLD should therefore be interpreted with awareness of potential definitional overlap. Abbreviations: ALT, alanine aminotransferase; BMI, body mass index; CAP, controlled attenuation parameter; FDR, false-discovery rate; FIB-4, Fibrosis-4 index; FPG, fasting plasma glucose; GGT, gamma-glutamyl transferase; HDL-C, high-density lipoprotein cholesterol; LDL-C, low-density lipoprotein cholesterol; LSM, liver stiffness measurement; MASLD, metabolic dysfunction-associated steatotic liver disease; TG, triglycerides.

**Table 5:** Factors associated with MASLD among HIV-negative controls.

Factor	OR (95% CI)	*p* Value	FDR-Adjusted *p*
**Panel A. FDR-significant univariable associations**			
Male sex	2.70 (1.30 to 5.61)	0.008	0.044
HOMA-IR, per 1-unit increase	1.37 (1.08 to 1.75)	0.011	0.049
Triglycerides, per 50 mg/dL increase	1.94 (1.37 to 2.74)	<0.001	0.003
HDL-C, per 10 mg/dL increase	0.41 (0.25 to 0.66)	<0.001	0.003
Alcohol use	3.21 (1.54 to 6.71)	0.002	0.015
**Panel B. Primary multivariable metabolic model**			
BMI, per 5 kg/m^2^ increase	1.12 (0.73 to 1.73)	0.597	—
HOMA-IR, per 1-unit increase	1.26 (0.97 to 1.64)	0.082	—
Triglycerides, per 50 mg/dL increase	1.66 (1.13 to 2.44)	0.010	0.041
HDL-C, per 10 mg/dL increase	0.60 (0.36 to 0.99)	0.047	0.094
**Panel C. Sensitivity model**			
Age, per 10-year increase	0.92 (0.63 to 1.33)	0.653	—
Male sex	3.58 (1.55 to 8.26)	0.003	0.008
Triglycerides, per 50 mg/dL increase	1.73 (1.19 to 2.52)	0.004	0.008
HDL-C, per 10 mg/dL increase	0.52 (0.32 to 0.85)	0.009	0.012

**Notes.** Odds ratios were estimated using mean bias-reduced logistic regression. Panel A shows univariable associations that remained significant after Benjamini–Hochberg FDR correction. The primary metabolic model included BMI, HOMA-IR, triglycerides, and HDL-C. The sensitivity model included age, sex, triglycerides, and HDL-C. The primary multivariable model included 147 participants and 44 MASLD events. Analyses used variable-specific complete cases without missing-outcome imputation. Repeating the analysis using the originally recorded MASLD classification preserved the direction and statistical significance of the triglyceride and HDL-C associations. Triglycerides and HDL-C are among the cardiometabolic criteria used to define MASLD; associations should therefore be interpreted with awareness of potential definitional overlap. All tests were two-sided. A dash indicates that an adjusted value was not reported for that estimate. Abbreviations: BMI, body mass index; CI, confidence interval; FDR, false-discovery rate; HDL-C, high-density lipoprotein cholesterol; HIV, human immunodeficiency virus; HOMA-IR, Homeostatic Model Assessment of Insulin Resistance; MASLD, metabolic dysfunction-associated steatotic liver disease; OR, odds ratio.

## Data Availability

The data presented in this study are available on request from the corresponding author. The data are not publicly available because they contain information that could compromise the privacy of people living with HIV.

## Supplementary Materials

The following supporting information can be downloaded at: https://www.mdpi.com/article/10.3390/ijms27198672/s1.
