# Supplementary material for: Baseline Liver Metabolic Health in Newly Diagnosed People Living with HIV Compared with HIV-Negative Controls: Cross-Sectional Findings from the Prospective MASHIV-TR Cohort
Source: Int J Mol Sci. 2026 Sep 28;27(19):8672. doi: 10.3390/ijms27198672 (PMC13664788; doi:10.3390/ijms27198672)
Supplement: Supplementary file 1 [file ijms-27-08672-s001.zip › ijms-4567141-supplementary.pdf]

Supplementary Materials

Baseline Liver Metabolic Health in Newly Diagnosed People Living with HIV Compared with HIV-Negative Controls: Cross-Sectional Findings from the Prospective MASHIV-TR Cohort

Manuscript ID: ijms-4567141 · International Journal of Molecular Sciences

Supplementary Table S1. FAST-related baseline comparisons and associated factors

Analysis	Comparison or predictor	Effect estimate or descriptive result	P value	FDR-adjusted P	Analysed n
**Panel A. Between-group FAST comparisons**					
Descriptive comparison	PLWH vs HIV-negative controls	0.10 (0.06–0.19) vs 0.13 (0.07–0.22)	0.065	—	250/158
Unadjusted continuous comparison	Hodges–Lehmann difference (PLWH minus control)	-0.02 (-0.03 to 0.00)	0.065	—	250/158
Adjusted continuous comparison	Model 1	-0.04 (-0.07 to -0.01)	0.003	—	408
Adjusted continuous comparison	Model 2	-0.04 (-0.06 to -0.01)	0.007	—	407
Adjusted continuous comparison	Sensitivity model	-0.02 (-0.05 to 0.00)	0.095	—	407
Overlap-weighted comparison	FAST score	-0.04 (-0.08 to 0.00)	0.071	—	Weighted cohort
Descriptive binary comparison	FAST ≥0.35, PLWH vs controls	20 (8.0%) vs 14 (8.9%)	0.759	—	250/158
Unadjusted binary comparison	Odds ratio, PLWH vs controls	0.89 (0.41 to 1.98)	0.854	—	250/158
Adjusted binary comparison	Model 1	0.87 (0.40 to 1.86)	0.712	—	408
Adjusted binary comparison	Model 2	0.86 (0.40 to 1.87)	0.709	—	407
Overlap-weighted binary comparison	FAST ≥0.35	0.84 (0.33 to 2.14)	0.721	—	Weighted cohort
**Panel B. Factors associated with continuous FAST score among PLWH**					
Spearman correlation	ALT, U/L	0.33	<0.001	<0.001	249
Spearman correlation	TG, mg/dL	0.21	0.001	0.021	240
Adjusted median regression	HIV RNA, per log10 copies/mL	0.01 (0.00 to 0.03)	0.048	0.279	248
**Panel C. Univariable factors associated with FAST ≥0.35 among PLWH**					
Bias-reduced logistic regression	ALT, U/L	1.04 (1.02 to 1.06)	<0.001	0.007	249

Analysis	Comparison or predictor	Effect estimate or descriptive result	P value	FDR-adjusted P	Analysed n
Bias-reduced logistic regression	TG, mg/dL	1.01 (1.00 to 1.01)	0.029	0.335	240
Bias-reduced logistic regression	CD4 count, cells/µL	1.00 (1.00 to 1.00)	0.032	0.335	250
Bias-reduced logistic regression	HIV RNA, log10 copies/mL	1.60 (0.91 to 2.82)	0.102	0.657	250
**Panel D. FAST score according to MASLD status**					
Within-group comparison	PLWH: MASLD vs no MASLD	0.178 (0.077–0.270) vs 0.094 (0.060–0.168)	0.003	—	250
Within-group comparison	HIV-negative controls: MASLD vs no MASLD	Distributional comparison	0.269	—	161

Notes. FAST scores are presented as median (Q1–Q3), binary phenotypes as n (%), continuous effects as differences with 95% confidence intervals, and binary effects as odds ratios with 95% confidence intervals. Model 1 was adjusted for age and sex; Model 2 additionally included BMI. The continuous sensitivity model used linear regression with HC3 standard errors. Binary models used mean bias-reduced logistic regression. Overlap weighting used the prespecified baseline propensity-score covariates. Continuous predictors were screened using Spearman rank correlation. FAST ≥0.35 associations were estimated using mean bias-reduced logistic regression. Benjamini–Hochberg FDR-adjusted P values are reported for exploratory analyses. For the HIV-negative control group only the P value was available, so subgroup medians are not reported. FAST ≥0.35 was used as a non-invasive phenotype and should not be interpreted as histologically confirmed MASH.

Abbreviations. ALT, alanine aminotransferase; BMI, body mass index; CI, confidence interval; FAST, FibroScan–AST score; FDR, false-discovery rate; HIV, human immunodeficiency virus; MASLD, metabolic dysfunction-associated steatotic liver disease; MASH, metabolic dysfunction-associated steatohepatitis; PLWH, people living with HIV; TG, triglycerides.

Supplementary Table S2. Covariate balance before and after propensity-score overlap weighting

Covariate	Unweighted SMD	Overlap-weighted SMD
Age	0.215	<0.001
Male sex	0.337	<0.001
Body mass index	0.431	<0.001
Current smoking	0.148	<0.001
Alcohol use: No	0.087	<0.001
Alcohol use: Yes	0.083	<0.001
Alcohol use: Missing	0.004	<0.001
Any comorbidity	0.161	<0.001
Physical activity: Exercise at least once per week	0.002	<0.001
Physical activity: No regular exercise	0.049	<0.001
Physical activity: Regular daily exercise	0.051	<0.001
Sleep duration: <8 hours/day	0.427	<0.001
Sleep duration: >8 hours/day	0.184	<0.001
Sleep duration: 8 hours/day	0.243	<0.001

Covariate	Unweighted SMD	Overlap-weighted SMD
Lifestyle pattern: Intensive work schedule	0.290	<0.001
Lifestyle pattern: Night-oriented lifestyle	0.044	<0.001
Lifestyle pattern: Regular lifestyle	0.203	<0.001
Lifestyle pattern: Sedentary lifestyle	0.044	<0.001
Dietary pattern: Fast-food-based diet	0.065	<0.001
Dietary pattern: High-protein diet	0.129	<0.001
Dietary pattern: High-protein diet, Fast-food-based diet	0.031	<0.001
Dietary pattern: Mediterranean diet	0.116	<0.001
Dietary pattern: Mediterranean diet, Fast-food-based diet	0.031	<0.001
Dietary pattern: Mediterranean diet, High-protein diet	0.046	<0.001
Dietary pattern: Vegetarian diet	0.038	<0.001
Dietary pattern: Vegetarian diet, High-protein diet	0.023	<0.001
Dietary pattern: Other	0.006	<0.001
Dietary pattern: Missing	0.017	<0.001
Education: High school	0.036	<0.001
Education: Middle school	0.091	<0.001
Education: Primary school	0.111	<0.001
Education: University	0.178	<0.001
Education: Other	0.004	<0.001
Education: Missing	0.008	<0.001
Triglyceride level	0.309	<0.001

Notes. Overlap weights achieve exact mean balance on every covariate included in the propensity-score model; weighted values are therefore reported as <0.001. An SMD below 0.10 is conventionally regarded as adequate balance. SMD, absolute standardised mean difference.

Supplementary Table S3. Effective sample size after overlap weighting

Group	Original n	Effective sample size
HIV-negative controls	161	76.6
PLWH	250	95.5

Notes. Overlap weighting attains exact covariate balance at the cost of statistical precision; the effective sample size quantifies that cost.
